# Obesity and Oral Health: Clinical Associations, Mechanistic Evidence, and Implications for Dental Care—A Narrative Review

**DOI:** 10.3390/biomedicines14102255

**Published:** 2026-10-06

**Authors:** Marzena Dominiak, Wojciech Niemczyk, Szymon Suwała, Tomasz Gedrange, Victor Geller

**Affiliations:** 1 Department of Dental Surgery, Faculty of Dentistry, Wroclaw Medical University, Krakowska 26, 50-425 Wrocław, Poland; marzena.dominiak@umw.edu.pl (M.D.); tomasz.gedrange@umw.edu.pl (T.G.); research@victorgeller.com (V.G.); 2 Department of Endocrinology and Diabetology, Faculty of Medicine, Collegium Medicum in Bydgoszcz, Nicolaus Copernicus University in Toruń, Skłodowskiej-Curie 9, 85-094 Bydgoszcz, Poland; 3 Clinic of Metabolic Rehabilitation, 22nd Military Hospital of Spa and Rehabilitation in Ciechocinek, Wojska Polskiego 5, 87-720 Ciechocinek, Poland

**Keywords:** obesity, oral health, periodontitis, oral microbiome, peri-implantitis, insulin resistance, adipokines, wound healing, jawbone, metabolic syndrome

## Abstract

Obesity is a heterogeneous multisystem condition characterized by chronic low-grade inflammation, oxidative stress, adipokine dysregulation, and insulin resistance; its effects on oral microbial homeostasis, tissue regeneration, and disease susceptibility vary with metabolic context and with the outcome examined. This narrative review synthesizes evidence on the mechanisms linking obesity to oral disease and on its implications for dental prevention, periodontal care, implant therapy, and oral surgery. MEDLINE via PubMed, Scopus, and Web of Science Core Collection were searched from January 2000 to 31 August 2026, with the final search performed on 10 September 2026. The strength of evidence differs markedly by outcome. Human epidemiological studies support an association with periodontitis; altered salivary flow and composition are reported mainly in cross-sectional studies. Proposed effects on osseous healing and jawbone remodeling rest substantially on experimental and systemic bone research, and specific marrow alterations remain poorly established. Obesity is associated with selected peri-implant inflammatory outcomes, whereas evidence on implant survival is less consistent; these findings establish neither impaired primary osseointegration nor a contraindication to implant therapy. Associations with caries appear largely attributable to shared determinants. The phenotype-aware framework proposed here is hypothesis-generating and requires prospective interventional validation.

## 1. Introduction

Obesity is a chronic multisystem condition characterized by excess adiposity, with a global prevalence that has risen substantially in recent decades [1]. Its clinical expression varies by fat distribution, inflammatory and metabolic profiles, and associated conditions, including type 2 diabetes, cardiovascular disease, and hypertension [2,3,4]. Obesity should therefore be considered a heterogeneous metabolic condition; BMI alone does not capture the factors potentially relevant to oral health.

Human observational studies and their syntheses support a correlation between obesity and periodontitis; however, findings exhibit variability across different populations and oral health outcomes [5,6,7,8]. The assessment of dental caries warrants a distinct interpretation due to the influence of shared dietary, behavioral, socioeconomic, salivary, medication-related, and metabolic factors, which complicate the attribution solely to adiposity [6,7].

Chronic low-grade inflammation, oxidative stress, altered immune responses, and endocrine dysregulation provide a biological foundation for investigating obesity-related changes in oral tissues [2,3]. Evidence concerning diabetes further elucidates mechanisms through which glycemic disturbances may influence oral microbial communities, immune responses, and tissue repair [9]. Adipokines, such as leptin, adiponectin, and resistin, have been examined as possible mediators of periodontal inflammation, tissue degradation, and regeneration [10,11,12]. Proposed interactions between the oral cavity and the gut, involving microbial dysbiosis, metabolic endotoxemia, and insulin resistance, supply additional mechanistic insight, though their causal contribution to obesity-associated oral disease in humans remains to be conclusively established [13,14].

Evidence concerning jawbone density and structure, marrow composition and adiposity, osseointegration, and surgical healing necessitates separate evaluation, because the available observations arise in three different clinical contexts that are not interchangeable. The first is skeletal morphometry in growing patients: pediatric imaging studies have documented differences in mandibular cortical and trabecular measurements between children and adolescents with and without obesity [15,16]. The second is marrow pathology in selected surgical populations, which is a different question in a different age group: a scoping review of fatty degenerative jaw lesions describes marrow abnormalities within specific clinical contexts, most of them unrelated to adiposity [17]. Neither line of evidence establishes uniform deterioration of jawbone integrity attributable to obesity, and neither was designed to do so. The third context is mechanistic and largely extraskeletal: altered vascular support, local hypoxia, and changes in mesenchymal stromal cell function are proposed as explanatory pathways, drawing on general bone research and on the jaw-lesion literature [17,18,19] and their operation in human obesity-related jawbone change remains to be verified directly. Observations of any of these three kinds cannot establish impaired primary osseointegration nor reliably predict implant failure; measures of peri-implant inflammation and implant survival are distinct endpoints [20].

Delayed bone repair has been observed following tooth extraction in a rat model of diet-induced weight gain [21]. Nonetheless, this experimental observation does not quantify postoperative healing risks in patients with obesity. Nutritional status adds a further modifying factor: micronutrient deficiencies have been documented within populations suffering from obesity, including children and adolescents [22,23,24]. When present, protein-energy malnutrition and specific micronutrient deficiencies may impair collagen synthesis, angiogenesis, and immune function, all of which are pertinent to oral wound healing [25]. Their existence and impact should be evaluated independently of adiposity and associated metabolic conditions.

This review addresses the knowledge gap regarding how well existing evidence distinguishes oral outcomes linked to adiposity from those associated with concomitant metabolic conditions and shared determinants. A related uncertainty concerns whether the proposed mechanistic explanations and biomarker findings provide a sufficient basis for specific clinical interventions in dental practice. The primary aim of this narrative review is to critically examine associations between obesity, related metabolic phenotypes, and oral health outcomes, as well as the proposed biological mechanisms. It also aims to evaluate the robustness and directness of the evidence supporting implications for dental care. The principal clinical focus includes periodontal and peri-implant diseases, oral wound healing, and jawbone characteristics relevant to implant therapy and oral surgical procedures. When an association is reported in participants with insulin resistance, metabolic syndrome, type 2 diabetes, dyslipidemia, vitamin D deficiency, or relevant medication exposure, the finding is attributed to the studied metabolic phenotype rather than to adiposity alone unless the study design permits those effects to be separated.

The review primarily addresses adults; pediatric findings are considered separately when they inform developmental or age-related differences. Obesity denotes excess adiposity as defined in the source study, whereas central obesity denotes abdominal adiposity and is treated as a distinct exposure. Metabolic syndrome denotes a defined cluster of cardiometabolic abnormalities; insulin resistance denotes impaired insulin action; and type 2 diabetes denotes established diabetes rather than insulin resistance alone. For this review, metabolically unhealthy obesity denotes obesity accompanied by adverse metabolic abnormalities such as insulin resistance, dysglycemia, dyslipidemia, or other components of metabolic syndrome. These conditions are analyzed separately and are not used interchangeably to define obesity.

Throughout this review, we differentiate between association and causation. Cross-sectional associations do not establish temporal order; longitudinal observational studies can document temporal sequences, although temporal ordering alone does not identify a causal effect. Statistical adjustments do not eliminate residual or unmeasured confounding, and attenuation after adjustment does not, by itself, demonstrate mediation. Causal interpretation is limited to the population, exposure, outcome, and experimental context evaluated, with the assumptions and limitations of the study design made explicit. Experimental mechanisms are not extrapolated to human oral disease as established causal effects without supporting human evidence. Heterogeneity in definitions of adiposity and oral disease further constrains causal attribution. Intermediate outcomes, such as biomarkers, inflammatory measurements, physiological parameters, and radiographic changes, are assessed independently from outcomes with direct clinical significance. Variations in these intermediate measures are not considered indicators of improvement or decline in survival, disease progression, postoperative complications, or other clinical endpoints unless such outcomes have been explicitly evaluated.

The review examines inflammatory, endocrine, microbial, and other experimental mechanisms insofar as they assist in interpreting the specified oral outcomes. Evidence related to dental caries, salivary dysfunction, halitosis, oral malignancy, sleep apnea, bariatric surgery, and pharmacotherapy for obesity is incorporated selectively, particularly when it elucidates oral risks, provides alternative explanations for observed associations, or informs dental treatment considerations; exhaustive accounts of these conditions or their management are not attempted. The contribution of the review lies in integrating mechanistic explanations with human clinical findings inside a framework that keeps adiposity, associated metabolic conditions, and shared determinants apart, and that appraises the evidentiary support for each proposed clinical implication separately from its biological plausibility. This narrative review is based on searches of PubMed/MEDLINE, Scopus, and Web of Science, supplemented by earlier seminal publications and reference-list searching; the search, eligibility assessment, selection process, and methodological appraisal are described in Section 2.

## 2. Review Methodology

### 2.1. Review Design and Literature Search

This study was designed as a structured narrative review supported by a comprehensive and reproducible literature search. Given the broad and mechanistically heterogeneous scope of the review, encompassing epidemiological, clinical, translational, animal, and in vitro evidence, a narrative synthesis rather than a formal systematic review and meta-analysis was considered appropriate. Nevertheless, a reproducible database-search strategy, predefined eligibility criteria, structured study selection, and an explicit hierarchy of evidence were applied to increase transparency and minimize selective evidence retrieval. The terms structured and comprehensive are used in this specific sense and are not claims of exhaustiveness. Structured refers to the predefined and reproducible architecture of the process described below: a single exposure block combined with eleven predefined thematic blocks, predefined eligibility and exclusion criteria, two-stage screening, and a predefined appraisal framework. Comprehensiveness was pursued by covering three bibliographic databases rather than one, by decomposing the review question into thematic blocks so that no clinical domain within the stated scope depended on a single query, by applying no language restriction beyond the availability of an English-language abstract, and by supplementing the electronic searches with backward citation searching of relevant syntheses and key primary studies.

A comprehensive electronic literature search was performed in MEDLINE via PubMed, Scopus, and Web of Science Core Collection. The principal search covered publications from 1 January 2000 to 31 August 2026. The final database search was performed on 10 September 2026. Earlier seminal publications were additionally considered when they represented primary evidence for mechanisms discussed in the review. Because the review encompasses several distinct dimensions of the obesity-oral health relationship, the search strategy consisted of a common obesity/metabolic exposure block combined separately with predefined thematic blocks covering: (1) periodontal disease; (2) peri-implant disease and osseointegration; (3) dental caries; (4) salivary dysfunction, xerostomia, and halitosis; (5) oral malignancy; (6) jawbone biology and marrow alterations; (7) oral wound healing and surgical complications; (8) oral and gut microbiome pathways; (9) insulin resistance, inflammation, oxidative stress, adipokines, hormonal and epigenetic mechanisms; (10) lifestyle, diet, physical activity, stress, oral hygiene, and socioeconomic determinants; and (11) bariatric surgery and obstructive sleep apnea.

Search terms were adapted to the indexing and field syntax of each database. Reference lists of relevant systematic reviews, meta-analyses, and key primary studies were additionally screened to identify publications not captured by the electronic searches. The core structure of every search was identical and took the form: exposure block AND thematic block AND date limit. The exposure block, for example, was rendered in PubMed/MEDLINE as (“Obesity”[Mesh] OR obes*[Title/Abstract] OR overweight[Title/Abstract] OR “Adiposity”[Mesh] OR adiposity[Title/Abstract] OR “central obesity”[Title/Abstract] OR “abdominal obesity”[Title/Abstract] OR “visceral adiposity”[Title/Abstract] OR “Metabolic Syndrome”[Mesh] OR “metabolic syndrome”[Title/Abstract] OR “Insulin Resistance”[Mesh] OR “insulin resistance”[Title/Abstract] OR hyperinsulin*[Title/Abstract]), and was combined in turn with each thematic block listed above. The complete database-specific strategies for all three databases and all eleven thematic blocks are provided in Supplementary Material S1.

### 2.2. Eligibility and Study Selection

Publications were eligible when they evaluated obesity, overweight, central adiposity, or a clearly defined obesity-associated metabolic phenotype in relation to oral disease, oral tissue biology, dental treatment outcomes, or biological mechanisms relevant to the review. Eligible human evidence included systematic reviews and meta-analyses, randomized and non-randomized interventional studies, prospective and retrospective cohort studies, case–control studies, and cross-sectional studies. Experimental animal and in vitro studies were eligible when they provided mechanistic evidence relevant to pathways discussed in the review. Narrative reviews were used primarily to identify relevant concepts and primary literature rather than as the principal evidence supporting clinical associations.

Definitions of obesity, overweight, central adiposity, metabolic syndrome, and insulin resistance established by the individual source studies were accepted because diagnostic thresholds varied according to population, age, study period, and guideline used. These exposures were nevertheless treated as distinct entities during evidence interpretation. Type 2 diabetes, dyslipidemia, vitamin D deficiency, obstructive sleep apnea, and post-bariatric status were considered metabolic or clinical modifiers rather than interchangeable definitions of obesity.

Eligible oral outcomes comprised periodontal disease, peri-implant disease and implant-related outcomes, dental caries, salivary dysfunction and xerostomia, halitosis, oral malignancy, oral wound healing and postoperative complications, jawbone structure and remodeling, marrow alterations, and other clinically relevant oral manifestations within the predefined scope.

Publications were excluded when they did not address an obesity-related exposure or relevant metabolic phenotype; did not report an oral, dental, craniofacial, or directly relevant mechanistic outcome; consisted solely of conference abstracts, editorials, letters, opinions, or isolated case reports without mechanistic relevance; lacked sufficient methodological information for interpretation; or duplicated data reported in another included publication. When multiple publications reported overlapping populations, the most comprehensive or methodologically informative report was prioritized, while companion publications were retained only when they provided distinct outcomes or mechanistic information.

All retrieved records were exported to a reference-management database and duplicates were identified electronically and subsequently verified manually. Screening was conducted in two stages. First, titles and abstracts were evaluated against the predefined scope and eligibility criteria. Potentially relevant publications then underwent full-text assessment. Reference lists of eligible systematic reviews, meta-analyses, and key primary studies were examined using backward citation searching to identify additional publications.

Each record was screened independently by at least two authors at both the title/abstract and the full-text stage; no record was excluded on the judgement of a single author. Disagreements concerning eligibility or interpretation were resolved by discussion between the two assessors, and a third author was consulted when consensus was not reached at that stage. Because this was a structured narrative review rather than a systematic review intended to generate an exhaustive pooled effect estimate, study selection additionally considered the directness of the evidence to the specific clinical or mechanistic question and avoidance of unnecessary duplication of evidence.

### 2.3. Methodological Appraisal

No uniform formal risk-of-bias instrument was applied because the review incorporated heterogeneous evidence types. Methodological limitations were evaluated qualitatively and used to calibrate the weight and directness assigned to individual findings and conclusions.

The study design, methodological limitations, directness, and certainty pertaining to the specific claim are regarded as distinct considerations. A systematic review is interpreted in accordance with the designs and limitations inherent in its contributing studies; a pooled association does not, however, translate into causal evidence through meta-analysis. Overlapping reviews are not regarded as independent confirmations. Particular emphasis is placed on direct human oral studies and relevant longitudinal or interventional evidence concerning clinical outcomes. Conversely, animal experiments, in vitro studies, and findings from systemic or non-oral tissues are utilized to inform biological mechanisms and hypotheses. Observations from human tissues, surgical procedures, and well-conducted mechanistic studies remain valuable sources of information, even in instances where an intervention has not been validated. Primary publications are employed to detail study-specific samples, measurements, comparisons, and numerical results; evidence syntheses of direct relevance are utilized to provide broader contextual understanding. The appraisal therefore asked different questions of different evidence types. For human observational studies it asked which confounders were measured and adjusted for, how the exposure and outcome were defined, and whether the design permitted temporal ordering. For evidence syntheses it asked what designs the pooled studies used, how heterogeneity was handled, and whether the included populations matched the claim being supported. For animal experiments it asked whether the model reproduced adiposity without dysglycemia and whether the tissue studied was oral or extraoral. For in vitro work it asked whether the cell type, stimulus, and concentration were physiologically plausible. The resulting judgement was expressed not as a score but as the weight and directness attached to the finding in the text and, for clinical statements, as the category assigned in Table 1, Table 2, Table 3 and Table 4.

For each primary interpretation, the exposure, population, biological compartment, comparator, and outcome are systematically identified. BMI and central adiposity are distinguished from conditions such as diabetes, insulin resistance, dyslipidemia, metabolic syndrome, nutritional deficiencies, and treatment exposures. Serum, saliva, gingival crevicular fluid, periodontal tissue, jaw marrow, and other tissues sampled are recognized as non-interchangeable. Findings from cross-sectional and longitudinal studies, as well as both adjusted and unadjusted estimates, along with observational and experimental effects, are carefully distinguished. Consideration is given to potential confounders such as smoking, diet, socioeconomic status, medication use, oral hygiene, and other relevant factors; statistical mediation is differentiated from causal mediation. Findings that are positive, nonsignificant, or conflicting are preserved. It is important to note that a nonsignificant result does not imply equivalence or the lack of an effect.

Three descriptors are used consistently when the role of obesity in a specific oral outcome is characterized, and they are not interchangeable. Risk indicator denotes an association observed in cross-sectional or other non-causal observational analyses, without any implication of effect. Potential modifying factor denotes evidence that adiposity or its accompanying metabolic abnormalities alter inflammatory response, disease severity, or treatment response, without establishing that adiposity initiates the outcome. Independent determinant denotes a relationship that persists in longitudinal or interventional evidence after adjustment for the principal competing explanations; on the evidence reviewed here this designation is not reached for any of the oral outcomes examined.

In Table 1, Table 2, Table 3 and Table 4, clinical considerations are categorized into three descriptive groups. Category A signifies established standard-of-care factors within the population, indication, and procedure, as supported by cited guidelines, consensus, or recognized clinical practice sources. Category B indicates reasonable considerations supported by observational evidence from human studies, including its synthesis and documented clinical or tissue observations. Category C pertains to emerging biomarkers, mechanistic strategies, and author-proposed interpretations or clinical extensions that necessitate validation. These categories describe the basis of an action or interpretation rather than a numerical quality score or a formal certainty rating. They index clinical readiness, that is, how far a stated consideration is already supported for use in practice, and not certainty of evidence in the sense of GRADE. GRADE terminology is deliberately not adopted, because a GRADE assessment presupposes a defined question, outcome, and body of comparable studies, conditions that a narrative synthesis spanning epidemiological, clinical, animal, and in vitro evidence does not satisfy. A single row may encompass A, B, and C components, with each source linked to the component it substantiates. It is possible for an observation to remain classified as B, while the phenotype framework derived from it remains classified as C; similarly, an established clinical action may be classified as A, while an obesity-specific extension of that action remains classified as C.

## 3. Biological Mechanisms Linking Obesity and Oral Disease

This section integrates the principal biological pathways relevant to periodontal and peri-implant disease, oral wound healing, and jawbone characteristics. Evidence measured directly in human oral tissues, oral fluids, or clinical oral outcomes is distinguished from findings derived from animal models, in vitro systems, systemic tissues, or non-oral organs. The latter are treated as mechanistic support or biological extrapolation and are not assumed to demonstrate that the same pathway operates in human oral tissues. Particular attention is given to the metabolic condition studied, biological compartment, experimental model, and the directness of the evidence. Mechanistic evidence is consolidated in this section and is not repeated in disease-specific sections unless required to interpret a particular oral outcome. Subsequent sections focus on disease-specific associations, clinical outcomes, and the extent to which the mechanisms summarized here may explain those findings.

### 3.1. Inflammation, Adipokines, and Hormonal Influences

Adipose tissue dysfunction in obesity can alter systemic inflammatory signaling and the balance of adipokines [26,27,28]. Systemic and local adipokine measurements are not interchangeable. Concentrations measured in serum, saliva, gingival crevicular fluid, and oral tissues represent distinct biological compartments and may show different associations with obesity and periodontal status. Associations between circulating biomarker concentrations and periodontal status do not demonstrate biological effects within periodontal tissues. Evidence for a local periodontal effect requires direct assessment in human periodontal tissues or cells and should be distinguished from systemic biomarker associations; experimental effects observed in human-derived cells in vitro do not, by themselves, establish that the same effect occurs in vivo. In the periodontium, dysbiotic biofilms initiate host responses involving IL-1β, TNF-α, and IL-6 [29,30]. These responses promote connective-tissue degradation and osteoclast formation through pathways involving receptor activator of nuclear factor-κB ligand [31,32]. Adipose-derived signals may modify this local response, although their contribution must be distinguished from the effects of periodontal infection itself. Adipokine and hormonal evidence is therefore considered together in this subsection to distinguish systemic endocrine and inflammatory associations from effects demonstrated directly in periodontal tissues or experimental models.

Akram et al. observed no significant differences in gingival crevicular fluid levels of leptin, adiponectin, or IL-6 between patients with periodontitis with and without obesity. However, pooled resistin concentrations were elevated in individuals with obesity [33]. Zhang et al. reported higher baseline serum concentrations of IL-6, TNF-α, and CRP, as well as increased gingival crevicular fluid TNF-α levels in obese patients. Serum leptin levels varied depending on the assessment time, with no significant difference observed at baseline [34]. It is important to note that local periodontal inflammation can influence these measurements. Additionally, the predominantly cross-sectional design of the studies, heterogeneous definitions of obesity, and inconsistent classifications of periodontal disease limit the ability to draw causal inferences.

Leptin exerts its effects via the leptin receptor, which is expressed in gingival tissues and periodontal ligament cells and can activate intracellular signaling pathways, including JAK2–STAT3 [35,36]. In vitro experiments show that IL-1β stimulation of human periodontal ligament cells increases leptin expression, and leptin exposure can enhance IL-6 and IL-8 production [35]. These findings illustrate cellular responses under experimental conditions and support a potential local inflammatory mechanism; however, they do not quantify its magnitude or establish its clinical significance in periodontal tissues in vivo. Adiponectin possesses insulin-sensitizing, anti-inflammatory, and osteoprotective properties; its decreased levels in obesity may diminish inflammatory regulation [26,27]. Studies involving oral epithelial and periodontal ligament cells describe effects on inflammatory and reparative responses [37,38,39], and experimental research also indicates that adipokine administration can inhibit osteoclast formation induced by bacterial lipopolysaccharides [40]. It is important to distinguish the effects of experimental adipokine administration from those associated with endogenous concentration levels.

Resistin expression is increased in periodontal cells and tissues [41]. Broader experimental evidence links resistin to inflammatory signaling and macrophage activity [42,43], while a separate study addresses its expression and regulation in osteoblasts and osteo-clasts [44]. Visfatin, also known as nicotinamide phosphoribosyltransferase, has inflammatory and immunomodulatory effects [45] and, in cultured human periodontal ligament cells, can stimulate MMP-1 and CCL2 expression [46] and alter regenerative responses in periodontal ligament cells [47]. Its expression is regulated by microbial challenge and biomechanical loading, which need not produce effects in the same direction [48]. Changes in the balance between RANKL and osteoprotegerin are proposed to connect adipokine signaling with osteoclastogenesis, although the contribution of individual adipokines re-quires direct verification. Chemerin promotes chemotaxis of immature dendritic cells and macrophages [49]. The secretion of IL-1β, IL-6, and TNF-α induced by chemerin has been observed in cultured human articular chondrocytes stimulated with recombinant active chemerin [50]; this is an extraoral tissue model, cited as mechanistic support rather than as evidence of chemerin activity in periodontal tissue. Research on experimental dyslipidemia suggests that chemerin is involved in alveolar bone loss in mice, while con-current in vitro studies show an increase in the resorptive activity of mature osteoclasts, without a corresponding increase in osteoclast formation [51].

Abood and Aldhaher documented elevated concentrations of salivary chemerin and visfatin in participants suffering from obesity and periodontitis when compared to nor-mal-weight individuals with periodontitis and periodontally healthy controls of normal weight [52]. Within the subgroup characterized by obesity and periodontitis, both salivary chemerin and visfatin exhibited positive correlations with plaque index, bleeding on probing, probing depth, clinical attachment level, and BMI [52]. Özcan et al. observed positive correlations between salivary chemerin and the gingival index, probing depth, as well as clinical attachment level, thereby supporting further investigation into its association with the severity of periodontal disease [53]. Elevated salivary visfatin levels and an increased abundance of subgingival Selenomonas noxia were also reported in individuals with overweight or obesity who have periodontitis, in comparison to healthy controls [54]. The same study proposed an exploratory salivary visfatin cutoff for differentiating these groups; however, this cutoff was not externally validated, nor did it establish additional clinical utility beyond standard periodontal examination [54]. Collectively, these findings endorse the necessity for mechanistic studies and further evaluation of chemerin and visfatin as prospective periodontal biomarkers; their implementation in routine diagnostics, risk prediction, or treatment decision-making necessitates further validation.

Experimental and transcriptomic data offer complementary evidence. Virto et al. documented increased alveolar bone loss and inflammatory responses in rats with diet-induced obesity alongside experimental periodontal disease [55]. Cai et al. identified 147 differentially expressed genes common to both obesity and periodontitis datasets, emphasizing FGR, LCK, FYB1, LY86, and P2RY13 in relation to macrophage-associated functions [56]. Although these findings support further exploration of overlapping path-ways, it is important to note that responses observed in animal models and shared gene expression patterns do not establish causality in human patients.

The periodontium functions as a hormonal target tissue: gingival fibroblasts, osteo-blasts, and periodontal ligament fibroblasts express sex steroid receptors, and gingival tissues are capable of metabolizing these hormones locally [57,58]. Sathish et al. review indicates approximately a threefold increase in the conversion of estrone to estradiol in inflamed tissue, along with more efficient progesterone metabolism in chronically in-flamed gingiva [57]. Consequently, local inflammation has the potential to alter hormonal exposure within periodontal tissues.

Female sex steroids can promote Th2 and regulatory T-cell responses; however, fluctuations in hormones during puberty, the menstrual cycle, pregnancy, and menopause have the potential to alter gingival inflammation without a corresponding increase in plaque accumulation [57,58,59,60]. The effects of androgens are contingent upon exposure levels and the experimental context. Testosterone supplementation has been shown to in-crease inflammatory infiltration; supraphysiological exposure has worsened ligature-induced bone loss; and prolonged depletion of testosterone has alleviated inflammatory resorption in experimental models. Additionally, findings from NHANES have linked higher testosterone levels with increased prevalence and severity of periodontitis in men [58]. In a cross-sectional analysis of the Multi-Ethnic Study of Atherosclerosis, higher free testosterone and lower sex hormone-binding globulin were correlated with an increased prevalence of reported periodontitis-related tooth loss among postmenopausal women, after adjusting for cardiometabolic and reproductive factors. Relevant associations in men did not reach statistical significance upon adjustment [61]. Detailed search, eligibility, se-lection, and methodological appraisal procedures are reported in Section 2.

These findings are pertinent to obesity as adipose tissue contributes to extragonadal aromatization, and metabolic dysfunction can influence circulating sex hormone-binding globulin levels, thereby affecting sex-steroid availability [18]. Sex hormone-binding globulin is primarily synthesized by the liver; experimental research associates increased hepatic lipogenesis with diminished expression of its gene [62]. These findings propose potential mechanisms whose effects are contingent upon local inflammatory exposure, tissue compartment, and endocrine context. For clinical interpretation, emphasis should be placed on directly measurable metabolic characteristics, including central adiposity, glycemic and insulin resistance markers, dyslipidemia, and defined metabolic syndrome, rather than on indirect developmental or endocrine proxies.

### 3.2. Oxidative Stress and Lipid Oxidation

Oxidative stress is hypothesized to connect metabolic dysfunction with periodontal tissue injury [2,63,64,65,66,67,68,69,70]. Reactive oxygen species are capable of damaging lipids, proteins, and nucleic acids, as well as altering inflammatory signaling pathways. In a case–control study, Atabay et al. reported increased oxidative damage and decreased antioxidant capacity in gingival crevicular fluid associated with obesity and periodontal disease [71]. These findings support the need for further investigation into localized redox imbalance but do not determine its temporal sequence or its independent role in periodontal destruction.

Oxidized low-density lipoprotein (oxLDL) is considered a candidate extrapolated mechanism rather than an established oral pathway. Experimental investigations utilizing mesenchymal stromal cells and osteoblasts demonstrate that oxLDL can hinder osteogenic differentiation through CD36-dependent suppression of canonical Wnt signaling and can influence osteoblast viability in a dose-dependent manner [72,73,74]. Its impact on bone re-modeling is not uniformly proresorptive, as experimental data also suggest inhibition of osteoclast differentiation and resorptive function under specific conditions [75]. These findings are primarily derived from in vitro studies and systemic skeletal models, rather than from human periodontal or jawbone tissues; therefore, the role of oxLDL in obesity-associated periodontal destruction, jawbone remodeling, or oral regeneration remains to be definitively established.

### 3.3. Insulin Resistance and Associated Metabolic Dysfunction

Insulin resistance is hypothesized to mediate interactions among adiposity, immune responses, and periodontal disease [76,77,78,79]. Impaired insulin signaling may coexist with altered vascular function and inflammatory responses, thereby providing potential path-ways through which metabolic dysfunction could influence periodontal tissues. Diabetes introduces additional mechanisms, including sustained hyperglycemia and advanced glycation end-products. Consequently, evidence from diabetes must be distinguished from evidence concerning adiposity or insulin resistance in the absence of diabetes [77,78].

Research on experimental periodontitis has documented inflammation within adipose tissue and decreased insulin sensitivity in animal models [80,81]. These findings encourage further examination of the impacts stemming from periodontal infection on metabolic tissues, thereby complementing the hypothesized effects in the reverse direction. Their interpretation relies on the specific model and exposure conditions; they do not definitively establish causality or determine its extent in human populations.

**Figure 1:**
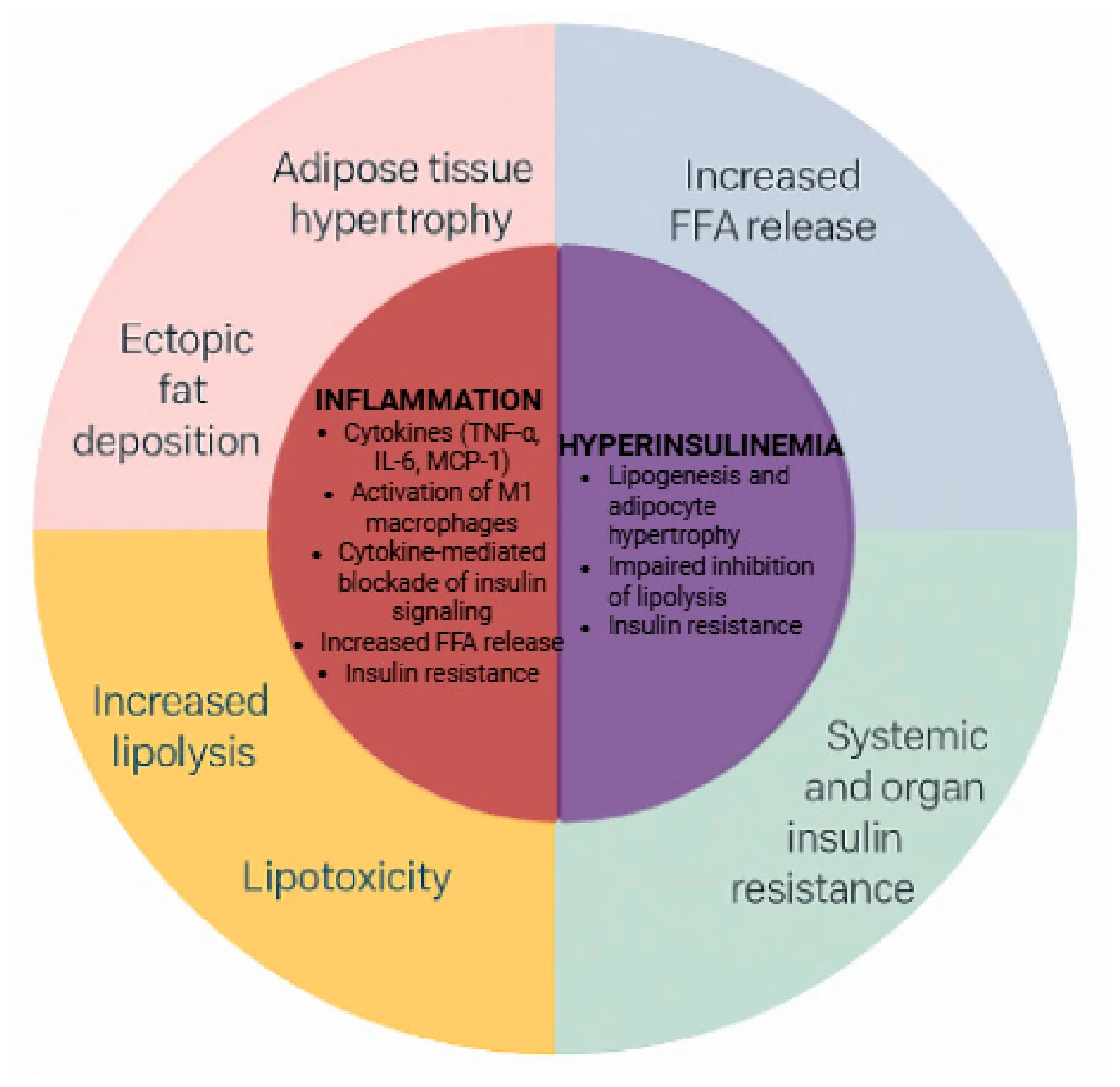
Insulin-free fatty acid-adipose tissue-inflammation axis.

Figure 1 summarizes proposed interactions among inflammation, hyperinsulinemia, adipose tissue dysfunction, ectopic lipid accumulation, and free fatty acid release.

Human evidence necessitates the differentiation of association, temporal sequencing, and intervention effects. While cross-sectional mediation analysis is capable of quantifying indirect associations within a specified model, it is inherently incapable of establishing temporal sequencing, biological mediation, or the proportion of a causal pathway functioning through insulin resistance. Consequently, such analysis does not confirm that insulin resistance precedes periodontal disease or that altering insulin resistance would influence the progression of periodontal conditions.

### 3.4. Oral Dysbiosis and Proposed Oral–Gut Pathways

Associations between adiposity and oral microbial composition differ across various populations, sampling sites, and periodontal conditions [82,83,84,85,86,87,88]. The existing research includes both adult and pediatric populations and does not demonstrate a consistent oral microbial signature associated with obesity. The systematic review focusing on pediatric populations particularly emphasizes the importance of interpreting microbial findings in the context of age, oral sampling site, and interstudy variation, rather than presuming that a singular taxonomic pattern is applicable across different populations [89]. Notably, summary taxonomic measures, such as the Firmicutes-to-Bacteroidetes ratio, should not be regarded as universal markers of obesity, given that their direction and magnitude vary across microbiome studies and populations. Factors such as periodontal status, diet, oral hygiene, metabolic comorbidities, and salivary conditions can influence interpretation. Furthermore, modifications in salivary composition and host responses have been suggested as contributors to the microbial environment [90]. Evidence pertinent to the oral–gut pathway can be categorized into three distinct types: Human observational studies that identify associations among oral microbial composition, adiposity, metabolic conditions, and systemic inflammatory markers—though these do not delineate causality or directionality; experimental models involving oral pathogens that demonstrate that specific microbial or periodontal exposures can induce systemic inflammatory and metabolic effects under controlled conditions—yet these effects should not be presumed to have the same magnitude or clinical importance in humans; and mechanistic hypotheses that synthesize these findings into proposed oral–blood and oral–gut pathways. In the absence of direct longitudinal or interventional evidence in humans, these pathways remain plausible biological hypotheses rather than confirmed mediators of obesity-related systemic inflammation.

Two proposed pathways establish a link between alterations in oral microbiota and systemic effects: the dissemination of microorganisms or their products via the circulatory system, and the passage of oral microorganisms into the gastrointestinal tract [14]. Evidence at the human strain level supports the transmission of microbes from the oral cavity to the gut, although this does not equate to demonstrating an association with intestinal pathology or obesity-related metabolic consequences [91]. The review con-ducted by Lam et al. explores mechanisms involving organisms such as *Porphyromonas gingivalis* and *Fusobacterium nucleatum*, disruption of the intestinal epithelial barrier, and microbial and immune interactions pertinent to inflammatory bowel disease [92]. The hypothesized subsequent processes encompass reactive T-cell responses, the production of cytokines such as IL-1β, IL-6, and TNF-α, as well as oxidative stress. The dissemination of microbial products and the persistence of immune activation may facilitate a feedback loop between oral and intestinal inflammation, although the processes of microbial trans-mission, intestinal pathology, and metabolic outcomes should be regarded as distinct phenomena [92,93,94].

Najeeb et al. reported correlations between oral dysbiosis and systemic inflammatory markers within a cross-sectional study involving 126 patients diagnosed with type 2 diabetes, cardiovascular disease, rheumatoid arthritis, or inflammatory bowel disease [95]. Oral dysbiosis was observed in over 60% of the participants, although the prevalence differed between the Results (61.1%) and the Abstract (64.3%). Subjects classified as having dysbiosis exhibited elevated levels of hs-CRP, IL-6, and TNF-α, with *p* < 0.001 for each comparison. Furthermore, reduced Shannon diversity indices were correlated with elevated concentrations of these markers, whereas inadequate oral hygiene was independently linked to dysbiosis [95]. The interpretation of these findings is constrained by conflicting descriptions of the microbial assessment: the Methods section states that 16S rRNA gene sequencing was conducted, whereas the Limitations section indicates that it was not performed. Such inconsistencies in reporting undermine confidence in the microbiological results. It is important to note that the cross-sectional nature of the associations precludes establishing a causal role of oral dysbiosis in systemic inflammation. Furthermore, their relevance to obesity and intestinal mediation remains indirect, as neither aspect was explicitly assessed [95].

Experimental models of oral pathogens have reported effects of periodontal infection or bacterial products on hepatic and adipose tissues, including inflammation, modifications in endocrine function, and disruptions in glucose and lipid metabolism [96,97,98,99]. These results illustrate biological effects observed under controlled experimental conditions; however, they do not confirm that oral dysbiosis induces similar systemic metabolic alterations in humans. The aspect of insulin resistance is addressed in Section 3.3. Rotaru et al. examine proposed connections between the oral cavity, gut, and liver involving nonalcoholic fatty liver disease, irritable bowel syndrome, and small intestinal bacterial overgrowth [100]. The hepatic findings are pertinent to current research on metabolic dysfunction-associated steatotic liver disease, although earlier studies employed the classification of nonalcoholic fatty liver disease [101]. The interpretation is complicated by shared factors such as obesity, insulin resistance, dietary exposures, and elevated systemic C-reactive protein. Alterations in immune responses and salivary conditions may promote oral dysbiosis, whereas periodontal cytokine release and endotoxemia are posited as pathways leading to hepatic and systemic metabolic inflammation. This is supported by experimental pathogen investigations and the oral–digestive synthesis [96,98,99,100].

The gut–immune–bone axis remains a biologically plausible extension rather than an established mechanism underlying obesity-associated jawbone disease. Experimental and systemic skeletal evidence indicates that reduced short-chain fatty acid availability, increased lipopolysaccharide exposure, and inflammatory signaling involving TNF-α, IL-6, and IL-17 can influence osteoclastogenesis and osteoblast differentiation [102]. Evidence on microbial regulation of immune-cell trafficking and bone-marrow niches also comes from non-oral contexts, including bone metastasis [103]. These findings support hypotheses about maxillary and mandibular remodeling, periodontal bone loss, implant outcomes, and regeneration, but they do not demonstrate that gut microbiome-mediated immune mechanisms alter human jawbone remodeling in obesity.

### 3.5. Epigenetic Regulation

DNA methylation, histone modification, and noncoding RNA activity constitute potential mechanisms through which metabolic and inflammatory conditions may influence periodontal gene expression [104,105]. Perri et al. observed elevated gingival expression of miR-15a, miR-22, miR-30d, miR-30e, miR-106b, and miR-142-3p in the cohort with obesity and periodontitis relative to the healthy cohort without obesity, although the increases did not uniformly reach statistical significance [104]. Bioinformatic analyses suggested roles for cytokine signaling, collagen metabolism, and lipid regulation. PPAR-γ was identified among the predicted targets; NF-κB and IL-6 were part of the broader proposed inflammatory regulatory framework. These findings highlight expression differences and potential targets rather than experimentally confirmed pathways linking individual microRNAs to periodontal destruction.

Xuan et al. investigated the histone demethylase JMJD3 and its interactions with adiponectin and IRF4 in macrophage regulation [105]. JMJD3 expression was found to be diminished in bone marrow and adipose tissue macrophages from mice subjected to diet-induced obesity. Adiponectin influenced the expression of JMJD3 and IRF4, as well as macrophage infiltration and polarization towards an anti-inflammatory M2 phenotype, and also mitigated periodontal bone loss in experimental conditions. A decrease in adiponectin may therefore contribute to impaired M2 responses and sustained pro-inflammatory M1 activity via altered epigenetic regulation. Nevertheless, JMJD3 also plays a role in lipopolysaccharide-induced inflammatory responses, and the effects of adiponectin appear to vary depending on the experimental setting. These findings support the exploration of macrophage-centered epigenetic interventions, although the clinical significance of this pathway in patients with obesity remains to be fully elucidated.

The regulation of vitamin D exemplifies a distinct tissue context. Experimental investigations involving the livers of mice subjected to a high-fat diet have correlated glutathione deficiency with changes in methylation and expression of genes governing vitamin D regulation [106]. The observed modifications encompassed genes related to vitamin D activation, such as CYP2R1, CYP27A1, and CYP27B1, as well as the vitamin D receptor and the catabolic enzyme CYP24A1. These findings imply a potential connection between redox imbalance and vitamin D metabolism; however, this process has not been demonstrated within human oral tissues based on the evidence provided here.

The significance of these regulatory mechanisms concerning the severity of oral diseases and the efficacy of treatment necessitates direct validation within suitable tissues and populations. Collectively, these mechanisms exhibit considerable variability in the robustness of direct evidence. Human oral tissue analyses and clinical observations support the local relevance of specific inflammatory, adipokine, microbial, and epigenetic findings. Conversely, numerous oral–gut, gut–immune–bone, and regulatory pathways are predominantly substantiated by animal studies, in vitro experiments, systemic data, or non-oral evidence. While such findings establish biological plausibility, they do not, in isolation, confer clinical utility, diagnostic or prognostic value, treatment indications, or enhanced patient outcomes. Therefore, proposed applications involving adipokine profiling, epigenetic mechanisms, microbiome modulation, or the gut–immune–bone axis should be considered as prospective future endeavors rather than established clinical practices. Their adoption requires direct human clinical or interventional evidence demonstrating reproducible benefits in diagnosis, prognosis, treatment selection, or patient outcomes.

## 4. Shared Determinants and Confounding

Diet, oral hygiene, physical activity, psychological stress, and socioeconomic conditions are shared determinants of obesity and oral disease. These factors can generate or strengthen an observed obesity–oral disease association even when adiposity has no direct biological effect on the oral outcome. They should therefore be considered potential confounders or common causes rather than automatically interpreted as mechanisms through which obesity produces oral disease. Accordingly, this section examines shared determinants as alternative explanations for observed associations. This distinction is particularly important for dental caries, for which the evidence reviewed here supports a predominantly common-risk-factor interpretation.

### 4.1. High-Sugar Dietary Patterns

High-sugar dietary patterns are recognized as shared determinants of obesity, metabolic dysfunction, and oral diseases. An increased intake of free sugars may contribute to excess caloric consumption and subsequent weight gain, while exposure to fermentable carbohydrates directly influences the development of dental caries [107,108,109]. Angarita-Díaz et al. reviewed evidence indicating that high-sugar diets modify the oral microbiota, including acidogenic and aciduric species such as *Streptococcus mutans* and *Lactobacillus* spp. [110]. Further microbial and dietary investigations corroborate alterations in biofilm composition, cariogenic potential, and oral ecological environments [111,112,113,114,115]. A systematic review examining free-sugar intake and periodontal disease, comprising 13 studies—12 of which were cross-sectional—primarily identified positive associations [116]. These findings support the exploration of periodontal contribution alongside the well-established pathways related to caries and weight management. While insulin resistance, systemic inflammation, and microbial alterations affecting periodontal tissues offer an integrated explanatory framework, the complete sequence remains unconfirmed by these dietary associations.

Prada et al. investigated perceptions regarding excessive sugar consumption: participants acknowledged associations with obesity, diabetes, and oral health issues but did not consistently recognize its broader systemic implications [117]. Evidence concerning actual consumption derives from dietary surveys. A review of 11 nationally representative European surveys identified significant sugar intakes, with sweet products and beverages serving as primary sources; estimates varied according to age and the definitions of total, added, and free sugars [118]. The WHO-oriented synthesis describes consumption exceeding recommended free-sugar limits and advocates for reducing intake to less than 10% of total energy, with a further reduction below 5% as a conditional recommendation [109]. These sources support public health concerns without treating perception studies as measures of dietary intake.

Emerging evidence indicates the potential benefits of direct periodontal intervention. In an exploratory randomized trial involving 21 patients undergoing periodontal therapy, a four-week period of abstinence from free-sugar was associated with reductions in body weight, visceral fat, and free-sugar intake, alongside an increased intake of vitamin C derived from fruit. Bleeding on probing was reduced in both study groups; however, the unadjusted between-group comparison was statistically nonsignificant, whereas an adjusted regression analysis indicated a preference for sugar avoidance [119]. This trial underscores the need for further investigation into dietary modifications during periodontal treatment, though it does not confirm sustained prevention of periodontal progression nor a treatment benefit specific to obesity. The reduction in sugar intake remains pertinent in the context of established weight management and caries prevention [109]. Additional clinical and research considerations include potential benefits for periodontal health, oral microbial homeostasis, and the broader cardiovascular–metabolic health context, with outcomes to be evaluated separately.

### 4.2. Oral Hygiene Behaviors

Evidence indicates correlations between obesity and oral hygiene, which may reflect behavioral patterns and biological interactions. Individuals with obesity may demonstrate increased plaque accumulation, gingival bleeding, and periodontal pocketing, while inadequate oral care may contribute to an inflammatory burden. In a clinical sample of 94 patients, Nijakowski et al. found that an approximate plaque index exceeding 40% was associated with roughly eightfold higher odds of obesity. A cleaning index above 4, calculated from brushing duration and frequency, was associated with approximately 85% lower odds of obesity; brushing twice daily for at least two minutes was also inversely associated with obesity [120]. These figures represent odds estimates from an observational clinical sample, rather than population-level risk reductions. Alam et al. investigated BMI, dietary and physical activity patterns, toothbrushing frequency and technique, and interdental cleaning among dental students [121]. Horizontal brushing was most prevalent in the underweight group; the study does not establish that horizontal or scrubbing techniques were specifically characteristic of participants with obesity. Shared psychosocial factors, refined carbohydrate intake, inflammatory context, and clustering of oral hygiene behaviors remain plausible explanations for associations between adiposity and oral health [120,121,122,123,124,125,126]. Enhancing oral hygiene remains clinically pertinent within the prevention and management of oral diseases in individuals with obesity, without implying that these observational findings demonstrate prevention of obesity itself.

### 4.3. Physical Activity, Stress, and Behavioral Clustering

Physical activity and psychological stress may influence oral health through immune regulation, inflammatory responses, and behavioral patterns. Medapati and Pachava synthesized associations between physical activity and oral health [127], while human observational studies reported lower prevalence of periodontitis among physically active participants [128,129]. A network meta-analysis of 123 randomized trials involving individuals with overweight or obesity found that exercise interventions affected systemic inflammatory biomarkers, with variations across different exercise modalities and markers, including CRP and IL-6 [130]. This evidence supports the impact of interventions on systemic inflammatory responses; however, it does not definitively establish that alterations in these biomarkers mediate periodontal health benefits. Anti-inflammatory and antioxidant responses remain plausible biological mechanisms warranting further investigation into direct oral health outcomes, in addition to the behavioral factors discussed below.

Population studies also examine relationships among physical activity, oral health measurements, and oral health behaviors [127,128,129,131,132,133,134,135]. Schmidt et al. observed that adolescents who consistently engaged in organized sports exhibited significantly lower caries experience and periodontal inflammation scores, while also demonstrating superior toothbrushing habits and fewer dietary risk factors. Furthermore, the study indicated that psychosocial stress, as assessed by the Strengths and Difficulties Questionnaire, adversely affected both oral health outcomes and oral hygiene practices, especially among individuals from socially disadvantaged backgrounds [136].

Stress-related neuroendocrine mechanisms constitute a complementary line of investigation. A systematic review and meta-analysis concerning salivary cortisol and periodontitis synthesized six cross-sectional studies; elevated cortisol levels were most prominently observed in cases of aggressive periodontitis, whereas the association with periodontal severity remained inconclusive [137]. A primary study examined psychological stress concomitantly with salivary cortisol, dehydroepiandrosterone, and chromogranin A in patients with gingivitis and periodontitis, thereby providing direct evidence for investigation of neuroendocrine markers in these oral conditions [138]. Conversely, Jiang et al. analyzed BMI, physical activity, and dental caries within the HUNT4 study; however, this research pertains to behavioral and caries-related evidence and does not investigate cortisol, attachment loss, or the stress-resilience pathway [139]. Additionally, Deshpande and Amrutiya offer further observational insights regarding obesity and oral health [140]. We propose that stress hormones, altered immune regulation, adipokine signaling, and shared behavioral patterns may interlink metabolic and periodontal vulnerability. Whether physical activity specifically diminishes oral susceptibility by modulating this stress pathway remains a hypothesis that warrants investigation through longitudinal or interventional studies.

Virtanen et al. provided behavioral evidence indicating that physically active adolescents are markedly more likely to brush their teeth twice daily and consume fewer sugary snacks compared to their inactive counterparts. This suggests that lifestyle clustering underpins much of the relationship between physical activity and oral health [141]. Collectively, these findings recognize physical inactivity and psychological stress as potentially significant behavioral and biological factors associated with oral disease. The inclusion of regular physical activity and stress management into comprehensive healthcare strategies is judicious, yet their incremental impacts on caries and periodontal health, along with the underlying mechanisms, necessitate outcome-specific investigation.

### 4.4. Socioeconomic Determinants

Socioeconomic disadvantage is linked to dietary and oral health conditions that may contribute to both weight increase and dental caries, including increased dependence on low-cost, energy-dense foods and sugar-sweetened beverages [142,143]. Subjective and objective socioeconomic factors also correlate with oral health, alongside psychosocial stress and behaviors such as oral hygiene, smoking, and dental visits [144]. A systematic review of longitudinal studies has associated earlier socioeconomic status with subsequent periodontal health, offering a temporal perspective on the periodontal component [145]. Evidence from children and adolescents further supports the interconnectedness of socioeconomic status, adiposity, and oral health [146]. It is proposed that these social and behavioral exposures interact with stress-related and inflammatory processes across the life course and potentially across generations. This comprehensive poverty–obesity–oral disease framework is an explanatory hypothesis rather than a definitive causal model. Interventions aimed at improving access to nutritious foods, primary care-based oral health services, and social resources merit assessment for their impacts on metabolic and oral health outcomes.

Collectively, these determinants offer alternative explanations for the observed associations between obesity and oral disease, and they limit the attribution of these associations to adiposity alone. Their contribution should therefore be considered prior to interpreting any observed association as evidence of a direct biological effect of obesity.

## 5. Oral Disease Phenotypes Associated with Obesity

The subsequent sections critically evaluate disease-specific human associations, clinically relevant outcomes, and their implications for diagnosis, prevention, and treatment planning. For each phenotype, interpretations distinguish associations observed directly with measures of adiposity from those that may reflect metabolic comorbidities, medication or treatment exposures, shared lifestyle and behavioral determinants, socioeconomic conditions, or other coexisting disorders. The strength and specificity of evidence are separately assessed concerning disease occurrence, severity, progression, treatment response, and clinically significant outcomes. Mechanistic plausibility is not considered as evidence that adiposity independently causes the phenotype. The pathways underlying mechanisms are not restated; where pertinent, their potential contribution is interpreted with reference to Section 3.

### 5.1. Periodontitis

This subsection analyzes evidence supporting both directions of the relationship between obesity and periodontitis: associations between adiposity and related metabolic phenotypes with periodontal prevalence, severity, and progression, as well as associations between periodontal disease or its treatment and systemic inflammatory and metabolic measurements. In this context, bidirectionality refers to the existence of reciprocal associations and biologically plausible interactions; it does not establish that obesity causes periodontitis, that periodontitis causes obesity, or that periodontitis causes metabolic disease. Section 3 delineates the shared biological pathways and corroborating experimental findings.

Among the phenotypes considered in this review, periodontitis has relatively substantial observational evidence linking it to adiposity. However, the specificity of this association is limited by variations in adiposity and periodontal definitions, as well as by coexisting metabolic, behavioral, and socioeconomic determinants. Evidence indicating that adiposity independently predicts periodontal progression is less consistent than evidence for cross-sectional differences in periodontal prevalence or severity. This inconsistency in longitudinal evidence should constrain the interpretation of obesity as an independent determinant of progression. Accordingly, the role assigned to obesity depends on the evidence available for the specific outcome. In cross-sectional or otherwise non-causal observational analyses, adiposity is regarded as a risk indicator associated with periodontal status. When evidence suggests that adiposity or its accompanying metabolic abnormalities modify inflammatory responses, disease severity, or treatment response, obesity is considered a potential modifying factor. When insulin resistance, dysglycemia, dyslipidemia, or other metabolic abnormalities cannot be discriminated analytically from adiposity, obesity is interpreted as part of a metabolic co-phenotype rather than as the independently responsible exposure. None of these designations establish obesity as a direct cause of periodontal progression.

Clinical and epidemiological studies have examined associations between adiposity and the prevalence, severity, and progression of periodontal disease. Suvan et al. synthesized evidence from 14 systematic reviews addressing obesity-related periodontal disease, responses to periodontal therapy, and inflammatory biomarkers [147]. Kim et al. reported a positive correlation between obesity and periodontitis in a systematic review and updated meta-analysis of observational studies [8]. Arboleda et al. examined epidemiological relationships between periodontal disease and various measures of adiposity, including BMI, waist circumference, and body fat percentage [148]. These findings support an association between adiposity and periodontal disease; however, the causal implications remain limited due to the observational nature of the underlying studies.

Longitudinal evidence addresses a distinct question. In the 10-year retrospective cohort reported by Charupinijkul et al., the unadjusted association between central obesity and periodontal progression did not persist after multivariable adjustment; the adjusted relative risk was 0.98, with a 95% confidence interval of 0.88–1.08 [149]. This is a progression result, rather than an estimate of periodontitis incidence, and the adjusted estimate does not support a positive independent association in that study. It also does not establish equivalence or the absence of an effect. Differences between unadjusted and adjusted findings suggest that accompanying metabolic, behavioral, or other shared determinants require consideration. These findings necessitate a differentiation among association, statistical mediation, and causation. Association refers to an observed relationship between an adiposity-related exposure and a periodontal outcome. Statistical mediation partitions an observed relationship within a specified model containing an intermediate variable. Causal mediation additionally requires justified assumptions about temporal order, confounding, and the relationships among exposure, mediator, and outcome. Cross-sectional mediation analysis does not inherently fulfill these criteria.

Jiang et al. examined cross-sectional NHANES data from 8473 participants evaluated during the years 2009 to 2012. They assessed HOMA-IR as a statistical mediator of the associations between adiposity and periodontitis [150]. Their mediation analysis estimated that the indirect component through HOMA-IR accounted for 63.44% of the modeled association involving body mass index and 36.77% of that involving waist circumference [150]. These percentages represent the decomposition of the fitted observational models and should not be interpreted as the proportion of a biological or causal pathway mediated by insulin resistance. Given that exposure, mediator, and periodontal status were evaluated concurrently, the analysis does not establish a temporal sequence, causal mediation, or that altering insulin resistance would impact periodontal outcomes.

Liu et al. reported an independent statistical association between general obesity and dental calculus, along with associations involving metabolic syndrome components such as abdominal obesity and hypertension [151]. These findings identify adiposity and metabolic abnormalities as correlates of calculus prevalence; however, they do not establish a causal relationship between obesity and calculus formation or periodontal inflammation. Possible explanations include metabolic or vascular influences on the oral environment, which necessitate further direct mechanistic investigation.

Menezes et al. examined systemic outcomes following periodontal therapy in patients with obesity and periodontitis [152]. Zhang et al. reported reductions in serum and gingival crevicular fluid IL-6, as well as in serum retinol-binding protein 4, subsequent to nonsurgical periodontal treatment in patients with obesity [34]. The literature summarized by Menezes et al. also encompasses alterations in inflammatory and metabolic biomarkers, including IL-6, TNF-α, hs-CRP, and HbA1c [152]. These findings suggest modifications in specific systemic inflammatory or metabolic parameters subsequent to periodontal intervention. However, they do not establish a reduction in cardiovascular events, incident metabolic diseases, or other clinically significant systemic outcomes. Therefore, improvements in biomarkers should not be construed as evidence that periodontal therapy diminishes cardiovascular or metabolic risk unless such clinical outcomes are explicitly assessed.

Periodontal findings subsequent to bariatric surgery exhibit variability based on clinical outcomes and the duration of follow-up. At the one-year mark, Arboleda et al. documented improvements in plaque index, bleeding on probing, and probing depth, whereas the clinical attachment level remained unaffected [153]. Additional cohorts and evidence syntheses report heterogeneous outcomes, including instances of periodontal deterioration during early postoperative follow-up [154,155,156,157,158]. Moreover, changes in oral hygiene practices, dietary intake, and nutrient absorption warrant careful consideration [136]. These observations do not substantiate the notion that weight reduction alone ameliorates periodontitis or leads to reversal of attachment loss.

### 5.2. Peri-Implant Tissue Outcomes, Osseointegration, and Implant Survival

The available clinical evidence supports the differentiation of five distinct implant-related outcome domains: implant survival, primary osseointegration, peri-implant mucositis, peri-implantitis, and marginal bone-level remodeling or loss. Implant survival pertains to the retention of the implant during the follow-up period and does not imply the absence of peri-implant disease. Primary osseointegration relates to the integration of bone at the implant interface and necessitates an outcome-specific evaluation rather than inference from subsequent inflammatory or radiographic findings. Peri-implant mucositis and peri-implantitis are separate inflammatory disease categories and should be reported as such only when the original study employed the corresponding diagnostic criteria. Marginal bone-level change constitutes a radiographic outcome and should not, by itself, be equated with peri-implantitis, impaired osseointegration, or implant failure; the terms marginal bone loss and marginal bone-level change are used interchangeably in this review for that radiographic outcome, and the wording of the source study is retained when its findings are reported.

A systematic review and meta-analysis involving 746 patients and 986 implants revealed no statistically significant difference in implant survival rates between individuals with overweight or obesity and those with normal weight. Neither study applied a case definition for peri-implant mucositis or peri-implantitis: the meta-analysis pooled survival together with inflammatory and radiographic parameters [20] and Vohra et al. reported plaque, bleeding on probing, probing depth, and radiographic bone level without diagnosing peri-implant disease [159]. Their findings are therefore treated here as inflammatory and radiographic measurements only. These findings support the consideration of obesity severity within the clinical context, without establishing a uniformly increasing gradient or a uniquely adverse Class III category. The observations pertain to implant survival, radiographic marginal bone alterations, and inflammatory parameters. Unless peri-implant mucositis or peri-implantitis was diagnosed using established criteria in the referenced studies, probing depth and bleeding on probing should not be equated with either diagnosis. A lack of statistical significance in survival comparisons does not imply equivalence. Studies involving obesity, metabolic syndrome, and type 2 diabetes should be interpreted based on the population actually examined [160]; the proposed biological pathways are discussed in Section 3.

Blanco et al. reported higher total and LDL cholesterol concentrations, increased white blood cell counts, and lower IL-10 levels in otherwise healthy participants with peri-implantitis compared to those with healthy implants [161]. Peri-implant probing depth was positively correlated with cholesterol levels and white blood cell counts, and negatively correlated with IL-10 levels. This cross-sectional study identifies associations with systemic measurements; however, it does not establish obesity as the causative factor nor demonstrate a bidirectional causal relationship.

The evidence summarized herein exhibits greater consistency concerning associations with peri-implant inflammatory measurements and marginal bone-level changes than with implant loss or survival. Nonetheless, direct evidence regarding primary osseointegration remains inadequate. Evidence pertaining to diagnosed peri-implant mucositis and peri-implantitis should be interpreted independently from studies that solely report probing depth, bleeding on probing, or radiographic bone-level alterations. The majority of current findings are observational and do not distinguish adiposity from concomitant metabolic diseases or local peri-implant determinants. Therefore, interpretation should be confined to the specific outcome under investigation. Associations with peri-implant inflammation or marginal bone loss do not imply impaired osseointegration nor establish an equivalent link to implant loss, and any findings in patients with diabetes should not be attributed solely to adiposity. Within this outcome-specific context, obesity may reasonably be regarded as a potential modifying factor for selected peri-implant outcomes rather than as a contraindication to implant therapy, recognizing that this characterization rests on predominantly observational evidence.

### 5.3. Dental Caries

The association between obesity and dental caries is complex and appears to be influenced by shared dietary, metabolic, microbial, and socioeconomic factors rather than a direct causal relationship. Both conditions are multifactorial, chronic diseases that share common risk determinants—most notably high intake of free sugars, poor dietary patterns, and socioeconomic disadvantage—that simultaneously contribute to weight gain and caries development [162,163]. Consequently, an observed correlation between obesity and caries may reflect common determinants such as sugar intake, socioeconomic status, oral hygiene, and access to preventive care, rather than an independent effect of adiposity itself. As such, the common-risk-factor model serves as the primary framework for interpreting the obesity–caries literature in this review. Factors including diet, socioeconomic circumstances, oral hygiene practices, salivary characteristics, medication exposure, and metabolic status may confound, mediate, or modify the observed associations and should be carefully considered prior to attributing caries susceptibility solely to adiposity.

Systematic reviews and meta-analyses have shown inconsistent results regarding the direction and magnitude of the obesity-caries link. Yu et al. conducted a meta-analysis including over 270,000 adults and reported no significant overall association between obesity and caries (OR = 1.06, 95% CI: 0.98–1.14), suggesting that previously observed relationships may have been confounded by diet and socioeconomic status [163]. Similarly, Piovesan et al. observed that the associations between obesity classifications and caries among preschool children were diminished after adjusting for socioeconomic circumstances and sugar consumption [162]. This finding supports the consideration of shared determinants; however, attenuation following adjustment does not, in itself, establish lifestyle factors as causal mediators.

Pediatric evidence exhibits heterogeneity. Silveira et al. concluded that longitudinal evidence regarding excess weight and caries was inconclusive [164], while Aprilia et al. reported a nonsignificant pooled association [165]. Conversely, a 2024 cumulative meta-analysis of 22 observational studies involving 40,673 participants identified a positive correlation between the extremities of BMI categories and early childhood caries, with an odds ratio of 1.44 and a 95% confidence interval of 1.16–1.78 [166]. These findings uphold both the evidence of a positive association and the uncertainty surrounding it; they do not establish an independent effect of adiposity nor confirm that the positive result was confined to a subgroup characterized by frequent sugar consumption and inadequate oral hygiene. Excessive exposure to fermentable carbohydrates remains a plausible shared determinant of obesity and dental caries. Bhoopathi and Tripicchio emphasized the importance of opportunities for integrated dental, nutritional, and behavioral prevention, given that childhood obesity and dental caries can persist into adulthood [167].

Salivary findings relevant to dental caries include assessments of flow rate and composition. An objectively reduced flow rate indicates hyposalivation, whereas alterations in pH, phosphate levels, peroxidase activity, inflammatory mediators, secretory IgA, or microbial presence constitute changes in salivary composition. Deng et al. synthesized differences observed between individuals with dental caries and those with overweight or obesity. The weight-related comparisons included lower unstimulated and stimulated salivary flow rates, decreased unstimulated salivary pH, reduced stimulated salivary phosphate levels, and decreased peroxidase activity in both groups. Additionally, higher levels of mutans streptococci, secretory IgA, and unstimulated salivary total antioxidant capacity were reported [168]. These findings pertain to processes involved in clearance, buffering, and remineralization; however, they do not establish a consistent causal relationship between adiposity and each salivary function. The elevated levels of IL-6 and TNF-α documented in that review should be attributed to the caries comparison rather than automatically to obesity [168].

Evidence specific to obesity-related cytokines is obtained from separate sources. Duffles et al. observed elevated salivary TNF-α in the obesity comparison, whereas the pooled salivary IL-6 comparison was not statistically significant [169]. Potempa-Jeziorowska et al. examined 102 children aged 7–10 years, including 28 with overweight or obesity and 74 with normal body mass, and reported increased salivary IL-6 in the group with excess body mass [170]. These findings support further investigation into inflammatory salivary differences, highlighting that results are contingent upon the marker, age group, and comparison. The extent to which the broader salivary profile is specifically attributable to adiposity, rather than metabolic conditions, diet, medication exposure, oral disease, or other factors, remains uncertain. Consequently, a proposed contribution to caries susceptibility is distinguished from established causation of caries progression.

Microbiological studies have reported correlations among adiposity, oral microbial composition, and the prevalence of acidogenic or aciduric bacteria such as *Streptococcus mutans* and *Lactobacillus* spp. These observations do not definitively demonstrate that adiposity independently causes a cariogenic microbiome. Nonetheless, these findings exhibit variability across populations, likely owing to differences in diet, fluoride exposure, and socioeconomic factors [171].

Collectively, the evidence supporting an independent correlation between adiposity and dental caries remains weak and inconsistent. A more coherent interpretation aligns with a common-risk-factor model, wherein obesity and caries share underlying determinants such as dietary sugar intake, socioeconomic disadvantages, oral hygiene practices, and access to preventive care. Additionally, alterations in salivary function, medication use, metabolic irregularities, and microbial variations may influence or mediate the observed associations; however, the reviewed evidence does not substantiate dental caries as a direct consequence of obesity. Therefore, preventative measures should concentrate on well-established determinants of caries and modifiable shared risk factors rather than obesity status alone. Biological mechanisms, including changes in salivary secretion, inflammatory responses, and microbiome dysbiosis, may further increase susceptibility to caries among individuals with obesity. As a result, comprehensive prevention strategies that integrate nutritional counseling, policies aimed at sugar reduction, and pro-motion of oral hygiene are vital for addressing both obesity and dental caries, particularly within socioeconomically disadvantaged populations.

### 5.4. Xerostomia and Salivary Dysfunction

This review distinguishes three salivary outcome domains. Xerostomia denotes the subjective perception of oral dryness and is assessed by patient-reported symptoms or questionnaires. Hyposalivation denotes objectively reduced salivary flow measured under defined unstimulated or stimulated conditions. Altered salivary composition denotes changes in the biochemical, immunologic, physicochemical, or microbial constituents of saliva and does not, by itself, establish reduced flow or subjective dryness. These outcomes may coexist but are not interchangeable. Evidence for an adiposity-specific association varies across these domains and remains limited and inconsistent. Xerostomia is prevalent among community-dwelling adults, affecting approximately 30–40% of this population, and coexists with various systemic conditions. Preliminary data suggest that obesity may contribute to dry-mouth symptoms and adversely affect oral health [172]. A more extensive study involving older adults revealed a significant burden of xerostomia, with strong correlations observed between the condition and hypertension, diabetes, as well as tooth mobility. Although measures such as BMI and waist-to-height ratio did not serve as independent predictors of xerostomia following adjustments, indices of adiposity were associated with patterns of tooth loss, thereby implying a relationship between metabolic and oral health even when dryness stems from multiple factors [173]. Accordingly, xerostomia in individuals with obesity should not be attributed to adiposity without considering alternative explanations, such as diabetes, medication exposure, sleep-disordered breathing, continuous positive airway pressure therapy, and other conditions that affect salivary function. In contrast, a clinic-based study of older Thai adults found that participants with overweight or obesity had higher clinical oral dryness scores and worse xerostomia questionnaire scores, and were nearly three times more likely to report poorer oral health-related quality of life [174]. These findings pertain to clinical signs of oral dryness and subjective xerostomia and should not be construed as evidence of objectively measured hyposalivation unless salivary flow has been assessed independently.

In summary, the extant multivariable evidence does not substantiate obesity as an independent etiology of xerostomia or hyposalivation. Correlations observed in populations with overweight or obesity should be considered in the context of age, diabetes, medication exposure, sleep-disordered breathing, treatment effects, and other factors influencing salivary function.

Mechanistic and translational evidence offer biological plausibility for effects on salivary gland structure and function [175]. Reported outcomes include changes in salivary flow and composition; however, these findings should be interpreted considering the measurement methods employed. Reduced stimulated or unstimulated salivary flow supports a diagnosis of hyposalivation, whereas biochemical or immunologic changes indicate altered salivary composition. Neither outcome should be used as a substitute for patient-reported xerostomia unless subjective dryness has been directly assessed. Sleep apnea-hypopnea syndrome may present an indirect pathway: a prospective case–control study reported increased xerostomia upon awakening among affected patients, with further morning dryness observed in individuals utilizing continuous positive airway pressure [176].

### 5.5. Halitosis

The evidence associating adiposity specifically with halitosis remains limited and heterogeneous. Existing studies evaluate various outcomes that should not be regarded as interchangeable. Self-reported halitosis reflects perceived oral malodor, whereas instrumental measurement of volatile sulfur compounds quantifies specific malodor-related compounds in exhaled oral air. An association with one outcome does not necessarily indicate a corresponding association with the other. These findings support an association that warrants further investigation but do not establish an independent effect of adiposity on oral malodor.

A national study of more than 68,000 Korean adolescents found that adolescents with obesity were more likely to report perceived halitosis than their normal-weight peers after adjustment for socioeconomic, behavioral, and oral-hygiene factors (OR = 1.22; 95% CI 1.06–1.42). These estimates pertain to self-reported perceived halitosis and should not be interpreted as evidence of objectively measured malodor or elevated concentrations of volatile sulfur compounds. The association was stronger in girls (OR = 1.60; 95% CI 1.17–2.20), suggesting possible hormonal or behavioral modification [177]. Participants with obesity also reported poorer oral hygiene, greater stress, and less frequent toothbrushing, indicating that systemic and behavioral determinants may combine to increase halitosis risk [177]. Persistence of an association after multivariable adjustment in an observational study does not confirm an independent causation, particularly when the outcome relies on self-reporting. Furthermore, residual confounding factors such as oral hygiene, dietary habits, psychosocial influences, periodontal health, and sleep-related variables may still be present.

A Erhamza et al. clinical study reported an association between BMI and halitosis [178]. A mechanistic interpretation requires separate evidence: microbial proteolysis and production of volatile sulfur compounds are established components of intraoral malodor, involving organisms such as *Porphyromonas gingivalis*, *Treponema denticola*, *Fusobacterium nucleatum*, and *Tannerella forsythia* [179]. We propose that obesity-related oxidative stress, insulin resistance, inflammatory signaling, gingival inflammation, and altered salivary conditions could modify this environment. This integrates metabolic and oral mechanisms discussed in Section 3, but the cited BMI association does not itself demonstrate that adiposity causes the complete cytokine–saliva–microbial sequence.

Bariatric surgery research further elucidates the obesity-halitosis link. A prospective longitudinal cohort by Alzahrani et al. evaluated 39 patients before and after sleeve gastrectomy and found a transient but significant increase in oral VSC levels (H_2_S and CH_3_SH) one month after surgery, coinciding with rises in *P. gingivalis* counts and worsening gingival indices [180]. These measurements offer objective evidence regarding alterations in selected volatile sulfur compounds; however, they do not confirm a corresponding change in perceived halitosis unless subjective symptoms have been evaluated separately. These findings document transient postoperative alterations in volatile sulfur compounds, periodontal measurements, and microbial findings; however, they do not isolate the independent effect of adiposity from the influences of surgery, rapid dietary changes, variations in oral hygiene, or concurrent metabolic alterations. Though halitosis symptoms tend to stabilize by six months postoperatively, these data underscore the need for oral health monitoring in multidisciplinary bariatric care.

Interpretation additionally necessitates the evaluation of oral hygiene, periodontal health, diet, psychosocial factors, xerostomia, and sleep-related conditions, which may influence both the association and the measured halitosis outcome. Reduced salivary flow can impair clearance and promote microbial putrefaction, while subjective dryness requires its own assessment [179]. Sleep-disordered breathing and its treatment offer possible indirect links: waking xerostomia and additional dryness during CPAP use have been reported in a prospective case–control study [176]. Their combined contribution to oral malodor is a proposed pathway and should not be regarded as an adiposity-specific effect. Findings regarding halitosis-related quality of life in patients with obesity pertain to a separate patient-reported outcome [181]. These distinctions uphold both the clinical significance of malodor and the hypothesis that salivary and sleep-related factors may influence its manifestation.

### 5.6. Oral Neoplasia and Tumor-Related Associations

Evidence connecting adiposity with oral neoplasia is heterogeneous and does not establish a consistent increase in incidence or prognosis associated with obesity across different oral malignancies. In a single-institution observational cohort of patients with surgically treated early-stage oral tongue squamous cell carcinoma, obesity was correlated with diminished disease-specific survival after multivariable adjustment, with an adjusted hazard ratio of approximately 5.0 [182]. This estimate pertains specifically to the studied population and clinical setting and should not be generalized to the incidence of oral cancer, other oral malignancies, or a causal relationship between adiposity and cancer progression.

Experimental evidence provides mechanistic plausibility; however, it must be distinguished from clinical evidence. In a 4NQO (4-Nitroquinoline 1-oxide) mouse model, diet-induced obesity altered oral tumor development and myeloid-derived suppressor cell activity [183]. A separate review discusses the role of obesity-associated myeloid-derived suppressor cells in cancer [184]. Experimental investigations of FTO-associated epitranscriptomic signaling, particularly related to arecoline-promoted oral carcinogenesis, do not constitute interventions on adiposity [185]. These sources uphold distinct lines of mechanistic evidence. They do not establish that adiposity triggers identical pathways, effect sizes, or clinical outcomes in human oral cancer. Human clinical evidence remains limited and substantially less conclusive.

Collectively, experimental models support the biological plausibility of adiposity-related influences on oral tumor biology. However, the existing human evidence remains inadequate to definitively establish an independent causal relationship with oral cancer incidence or progression. Consequently, the current evidence does not support implementing obesity-specific diagnostic, prognostic, or therapeutic strategies. Human associations ought to be interpreted within the context of established behavioral, clinical, metabolic, and treatment-related determinants.

### 5.7. Obstructive Sleep Apnea (OSA) and Oral Health

Obesity constitutes a major risk factor for OSA; however, oral findings in individuals with OSA should not be solely ascribed to adiposity. Excess adipose tissue may contribute to upper airway narrowing and collapsibility [186]. Factors such as intermittent hypoxia, sleep fragmentation, mouth breathing, treatment exposure, and craniofacial anatomy offer distinct potential pathways impacting oral health [187]. The airway-volume study referenced here did not establish an association with periodontal risk [188]. This null result should be distinguished from the broader mechanistic hypotheses, and the respective contributions of adiposity, OSA, and its treatment warrant separate evaluation.

OSA has been linked to specific outcomes related to caries, gingival, periodontal health, and oral health-related quality of life, although the populations studied, measurement techniques, and potential confounders vary across the literature [187,188,189,190,191]. Mouth breathing and alterations in salivary composition may contribute to plaque accumulation and oral dryness. Direct clinical observations support waking xerostomia and additional dryness during CPAP therapy [176]. Mechanistic insights include intermittent hypoxia and inflammatory signaling pathways, such as NF-κB-related pathways; however, cytokine levels should be interpreted within specific tissue compartments. Nizam et al. examined salivary inflammatory markers, including IL-6, but salivary TNF-α was not consistently elevated across their studies [192,193]. Gamsiz-Isik et al. evaluated levels of IL-1β in gingival crevicular fluid and hs-CRP in serum within a case–control study concerning OSA and periodontal disease [194]. These findings support investigating both local and systemic inflammatory variations without presuming that cytokines such as IL-6, IL-1β, and TNF-α are uniformly elevated across different biological compartments. Obesity-related adipokine dysregulation may amplify these inflammatory responses.

Craniofacial and occlusal features, including mandibular retrognathia, maxillary constriction, and altered tongue posture, may contribute to airway obstruction and influence orthodontic and restorative planning [195]. Studies have investigated sleep bruxism and masticatory muscle activity in relation to OSA [196]. Additionally, a systematic review has reported associations between bruxism and implant failure or mechanical complications of implant-supported restorations [197]. Potential outcomes such as tooth wear, temporomandibular symptoms, and excessive load are clinically relevant; however, the complete pathway from obesity through OSA and bruxism to these outcomes remains inadequately established in the current literature. Associations between obesity and oral findings within OSA populations may therefore be influenced or modified by factors such as the sleep disorder itself, mouth breathing, CPAP therapy exposure, craniofacial morphology, bruxism, and other comorbid conditions. An adiposity-specific effect warrants analytical differentiation of these factors.

## 6. Jawbone Alterations Associated with Obesity

### 6.1. Bone Density, Architecture, and Remodeling

Imaging studies conducted within younger populations report inconsistent relationships between adiposity and mandibular measurements. Yasa et al. documented greater mandibular cortical thickness and elevated panoramic mandibular index values in adolescents characterized by overweight or obesity [15]. Temur et al. examined 34 children and adolescents with obesity alongside 39 without obesity, discovering lower bilateral panoramic mandibular index values within the obesity cohort. Their results did not demonstrate statistically significant differences between the groups concerning mandibular cortical width, mandibular cortical index, or fractal dimension at the specified regions [16]. Consequently, the findings of Temur et al. should not be interpreted as definitive evidence of reductions in cortical width and fractal dimension. These observations suggest that cross-sectional associations are variable across pediatric populations and depend on specific imaging parameters.

Possible explanations encompass the competing effects of functional loading, anabolic leptin and insulin signaling, as well as the detrimental impacts of inflammation, oxidative stress, and modified adipokine balance on remodeling processes [18]. A biphasic response, characterized by initial cortical thickening followed by structural deterioration, remains a hypothesis. The referenced studies do not demonstrate a longitudinal transition from cortical thickening to cortical thinning or mineral depletion. Testing such a trajectory necessitates repeated measurements within the same population, and its significance concerning adult jawbone, implant, and surgical outcomes warrants separate investigation.

### 6.2. Marrow Adiposity and Local Degenerative Changes

Marrow adiposity, vascular support, and bone quality are discrete yet potentially interactive attributes of the jawbone microenvironment. An increase in marrow fat may influence the relative proportions of hematopoietic and adipose tissues, whereas diminished vascular support can compromise oxygen delivery and local tissue viability. The quality of bone is also contingent upon trabecular architecture, cortical structure, cellular activity, mineralization, and remodeling capacity. Currently, evidence directly associating these characteristics with obesity in human jawbones remains limited, and findings derived from systemic skeletal research should not be presumed to be directly applicable to the maxilla or mandible.

Dominiak et al. reviewed the literature and employed the term “fatty degenerative osteonecrosis of the jaw” to characterize jaw lesions marked by fatty marrow alterations, liquefied adipocytes, necrotic trabecular bone, and reported changes in local inflammatory mediators [17]. The concept remains controversial and lacks standardization; therefore, the terminology should not be interpreted as indicative of a diagnostic entity with equivalent nosological status to conditions such as periodontitis, medication-related osteonecrosis of the jaw, or conventional osteonecrosis. Furthermore, marrow adiposity and osteonecrosis are regarded as separate pathological entities: an increase or alteration in marrow fat does not, in isolation, confirm bone necrosis. Likewise, the term “silent inflammation” reflects terminology used within this body of literature rather than representing an independently validated diagnostic category.

The evidence reviewed herein, therefore, supports the investigation of fatty and degenerative marrow changes; however, it does not establish fatty degenerative osteonecrosis of the jaw as an obesity-associated diagnosis, a standardized case definition, or a validated disease category. Accordingly, gross appearance should not be used to distinguish among marrow adiposity, ischemic change, osteonecrosis, or other degenerative alterations without appropriate histopathological or other outcome-specific assessment. Alternative explanations for abnormal marrow or bone appearance should, therefore, be considered, including local anatomical and remodeling variation, metabolic or nutritional abnormalities, previous treatment exposure, and other causes of altered bone quality. Visual appearance alone cannot determine which of these explanations is operative.

**Figure 2:**
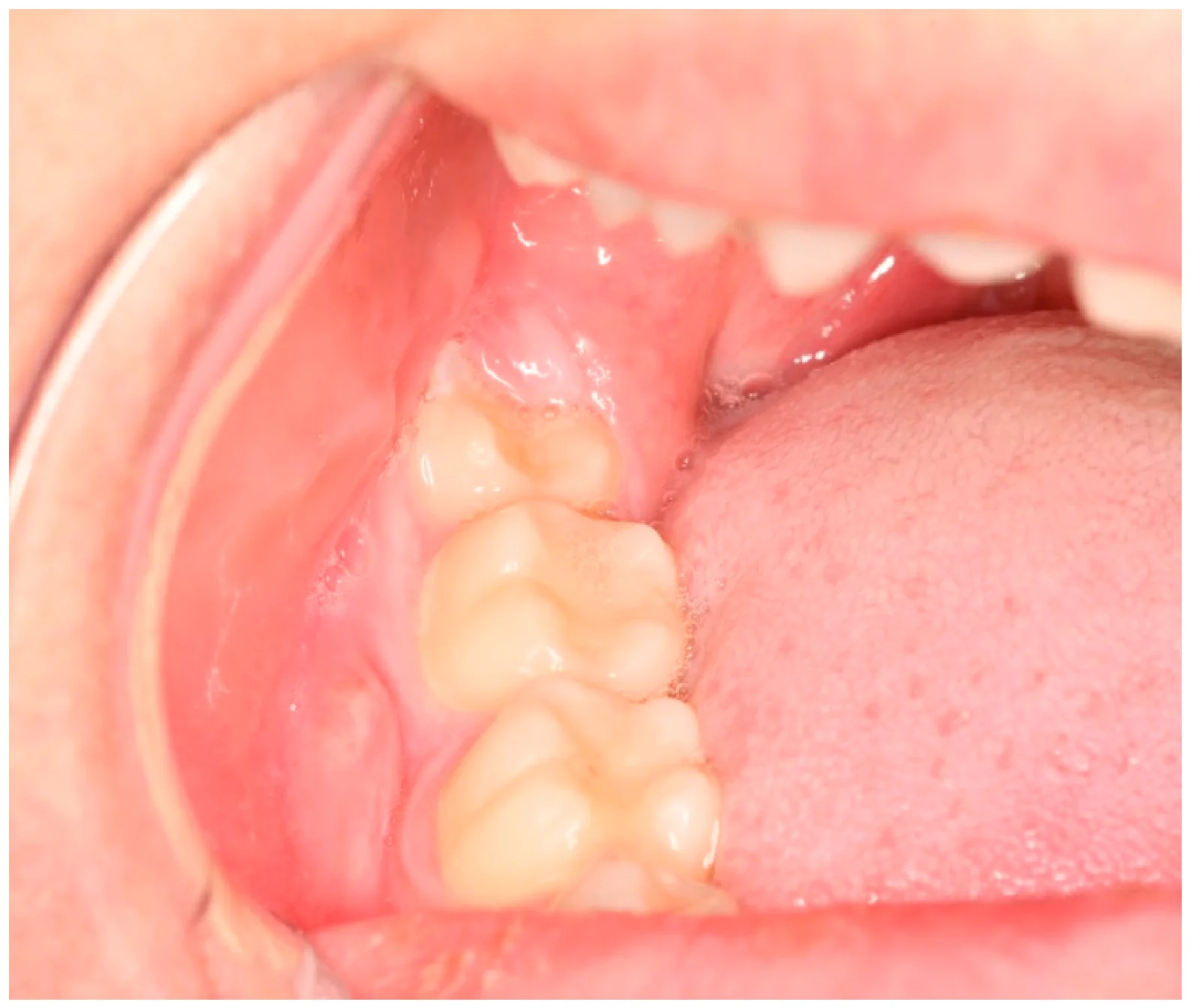
Intraoperative view of the mandibular vestibular region in a patient with obesity (class I, BMI 30.0–34.9 kg/m^2^), showing tissue of macroscopically fatty appearance adjacent to a thinned cortex. The image serves a descriptive purpose and does not establish marrow steatosis, osteonecrosis, or fatty degenerative osteonecrosis of the jaw.

**Figure 3:**
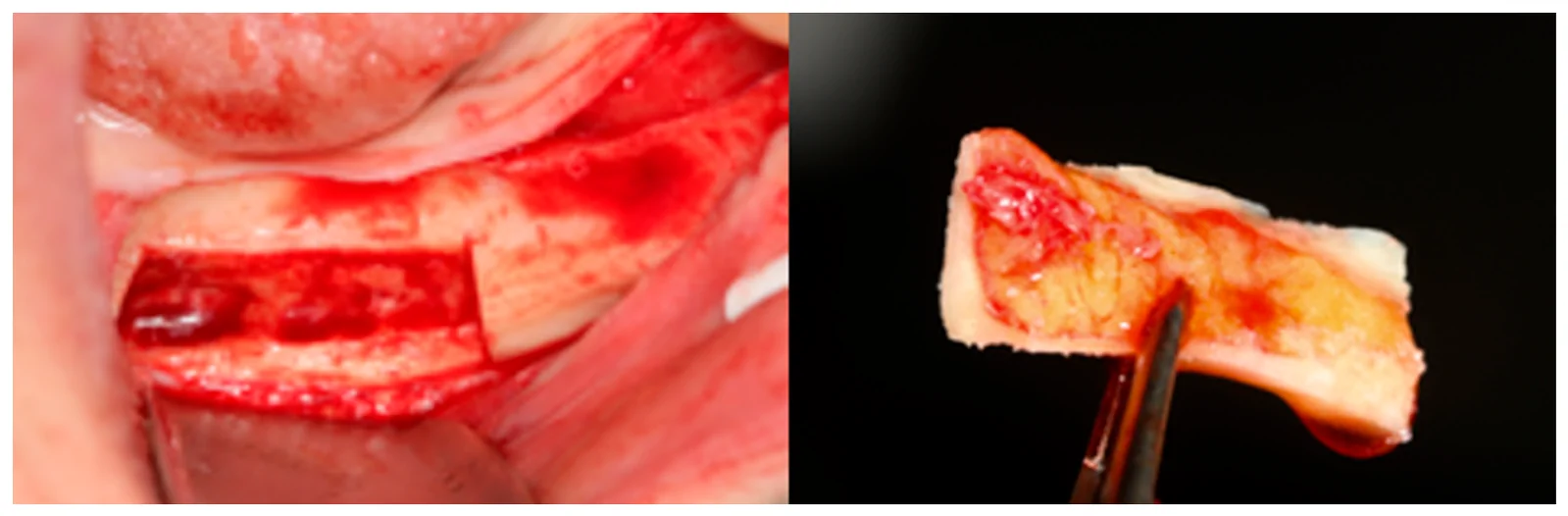
Intraoperative view during bone harvesting for augmentation in a patient with obesity (class II, BMI 35.0–39.9 kg/m^2^), showing marrow of macroscopically fatty appearance. The image is descriptive and does not establish the histological composition of the marrow.

**Figure 4:**
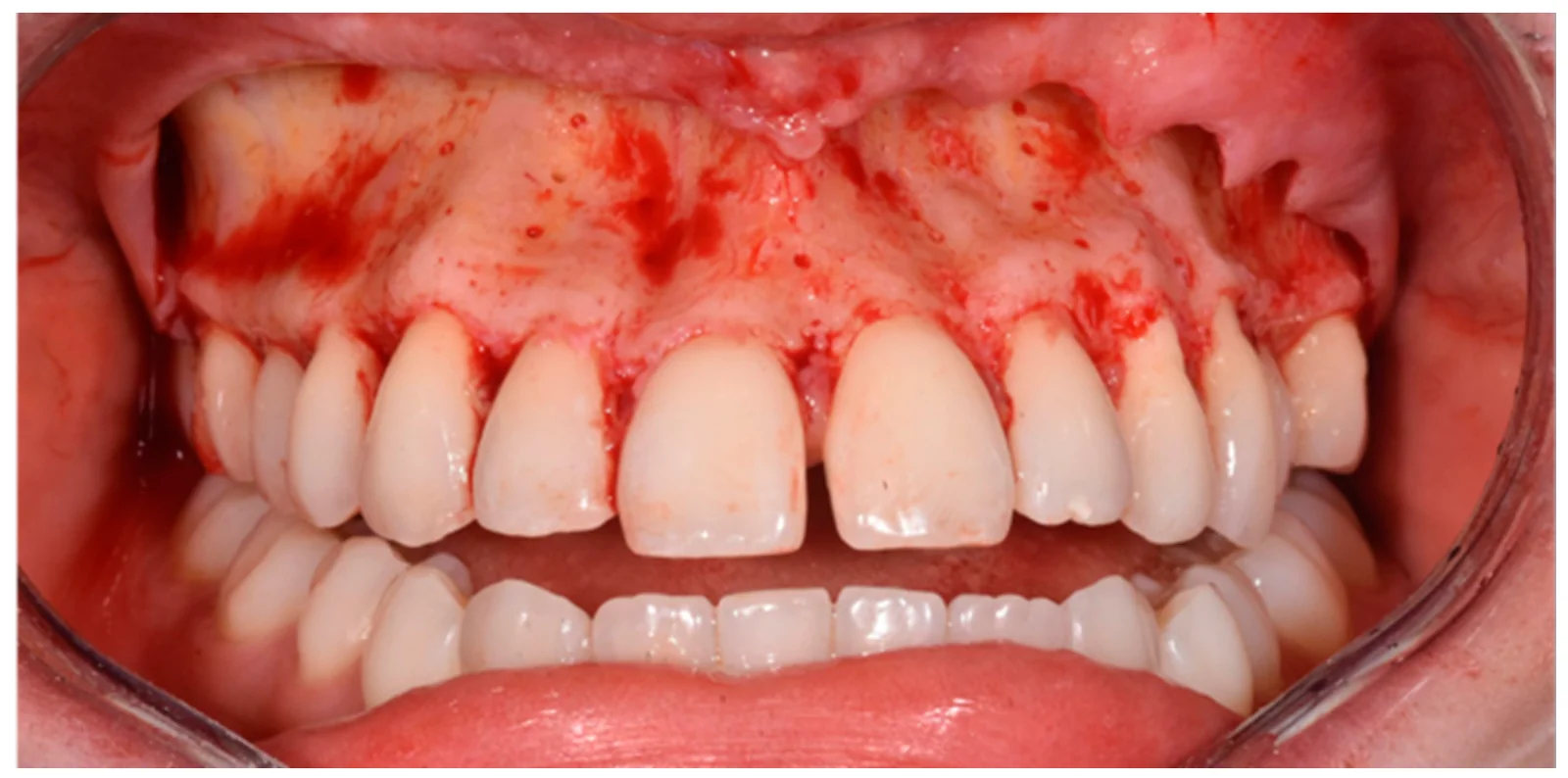
Intraoperative yellow discoloration of the bone on the vestibular aspect of the jaw in a patient with obesity (class II, BMI 35.0–39.9 kg/m^2^). The macroscopic appearance is descriptive and does not confirm fatty degeneration or osteonecrosis histopathologically.

**Figure 5:**
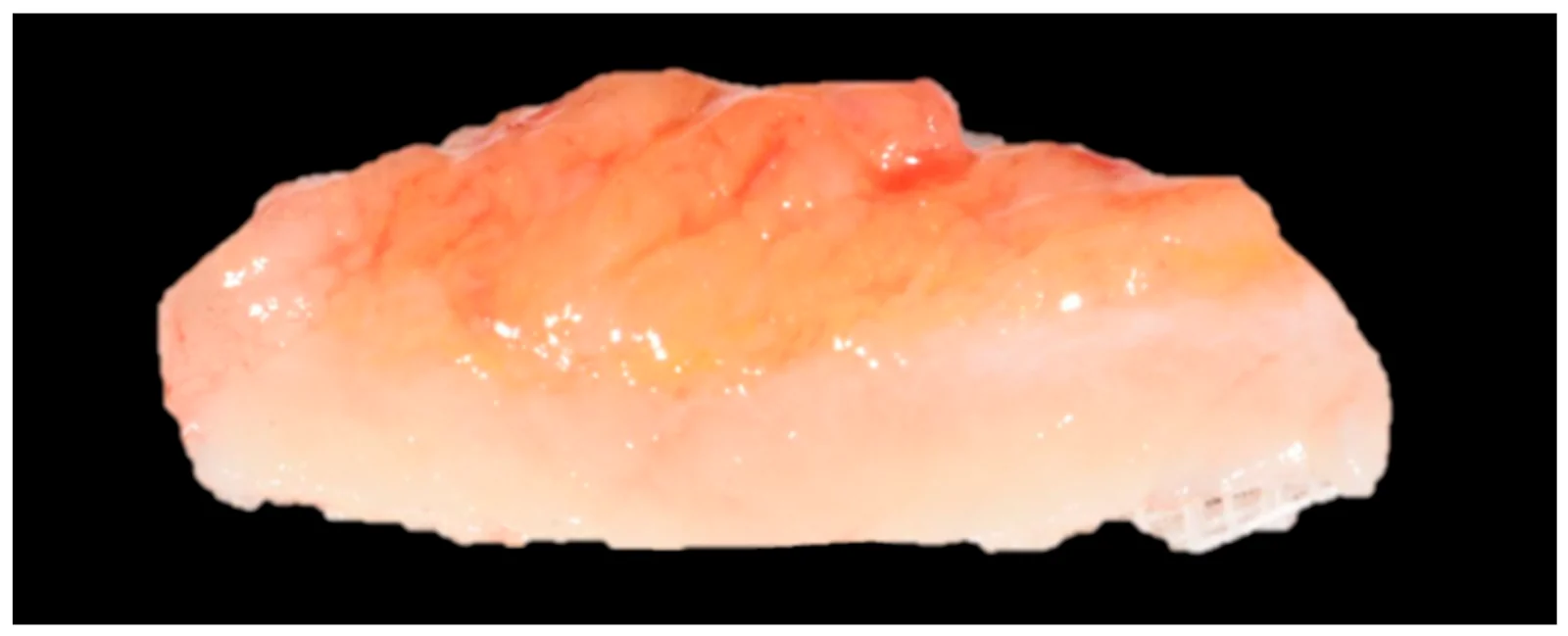
Macroscopic appearance of palatal donor tissue obtained during connective tissue harvesting in a patient with obesity (class II, BMI 35.0–39.9 kg/m^2^). The photograph is presented as a clinical illustration; the tissue was not examined histologically.

The clinically relevant focus is therefore on measurable marrow composition, vascularity, structural integrity, and remodeling rather than on assigning a specific diagnosis based on gross appearance. Figure 2 provides a clinical illustration of fatty tissue in the mandibular vestibular region, associated with cortical thinning and marrow changes. Gross appearance alone cannot establish histologic marrow adiposity, demonstrate osteonecrosis, or diagnose fatty degenerative osteonecrosis of the jaw. The image is descriptive and should not be interpreted as histopathological confirmation or as evidence of an obesity-specific lesion (the clinical photographs presented in this review were obtained during routine surgical care and were selected retrospectively for illustrative purposes). Potential surgical consequences of altered marrow composition and vascular support are considered in Section 6.5, while interactions with vitamin D deficiency and therapy-related osteonecrosis are considered in Section 6.4. The clinical photographs presented in this review (Figure 2, Figure 3, Figure 4 and Figure 5) were obtained during routine surgical care and selected retrospectively for illustrative purposes. All depict patients with obesity, but no standardised metabolic characterisation was performed at the time of surgery and these data could not be reconstructed retrospectively; none of the depicted tissue was examined histologically. The images therefore establish neither the prevalence nor the diagnostic specificity of the findings shown, their histopathological identity, nor a causal association with obesity.

### 6.3. Craniofacial Growth and Functional Adaptation

Developmental evidence indicates that nutritional patterns and adiposity may be linked to craniofacial morphology; however, these are considered distinct exposures. Boo Gordillo et al. conducted a pilot study on early feeding and jaw development; it should not be construed as direct evidence of caloric excess or obesity-related metabolic dysregulation [198]. More specifically, a systematic review of 10 cross-sectional studies involving 1383 children and adolescents examined the relationship between overweight or obesity and craniofacial morphology, reporting differences in selected craniofacial measurements [199]. These findings support further investigation into adiposity-related developmental associations, with consideration given to age, maturation, and nutritional status as separate factors.

At the molecular level, Zhao et al. investigated FTO, an m^6^A RNA demethylase, in the context of osteogenic differentiation of suture stem cells and the remodeling of the midpalatal suture during experimental rapid maxillary expansion. FTO inhibition led to a reduction in the mRNA expression levels of Runx2, Bmp2, and Col1a1, as well as a decrease in RUNX2 protein levels, thereby supporting its involvement in osteogenic differentiation and suture remodeling in both rat models and cellular systems studied [200]. It is important to note that this experiment provides mechanistic evidence; however, the presence of FTO within the pathway does not, by itself, establish that childhood obesity directly causes the craniofacial alterations observed.

### 6.4. Vascular Compromise and Pathologic Bone Changes

Wessel et al. reported an association between obesity and osteonecrosis of the jaw in a single-center, matched case–control study involving patients with cancer [201]. Among 30 cases and 150 controls, obesity was found to be associated with osteonecrosis in a conditional logistic regression model that accounted for zoledronate exposure, smoking, and metastatic disease, yielding an OR 16.61 (95%CI: 1.80–153.71) [201]. This imprecise estimate is specific to the particular treatment context studied and does not establish obesity as an independent predictor of medication-related osteonecrosis of the jaw (MRONJ) universally. A subsequent matched case–control study identified bisphosphonate type and bevacizumab exposure as significant in its final multivariable model [202]. Baba et al. further investigated the incidence and risk factors of MRONJ in a retrospective cohort comprising 91 patients with advanced cancer receiving high-dose antiresorptive therapy [203]. These investigations underscore the importance of evaluating medication history, disease status, and local oral factors within the treated population. Variations in reported predictors, or the omission of certain factors in specific models, do not imply that an effect of obesity is absent.

Experimental evidence addresses a different question. In the study by Lima et al., rats with diet-induced obesity that underwent zoledronic acid exposure and molar extraction developed larger areas of mandibular necrosis than animals subjected to the osteonecrosis protocol without obesity [204]. Adipocyte enlargement and dilated vessels consistent with blood stasis were also observed in the sampled adipose tissue. These findings support biological plausibility under controlled experimental conditions but do not demonstrate that obesity independently predicts MRONJ in humans or quantify its clinical contribution.

Potential interactions involving medication-related osteonecrosis encompass endothelial dysfunction, chronic inflammation, impaired oxygen delivery, and diminished remodeling capacity, combined with exposure to antiresorptive agents and local surgical trauma. Vitamin D deficiency introduces an additional nutritional pathway through impaired mineralization, secondary hyperparathyroidism, and, when severe enough, osteomalacia. The review conducted by Leizaola-Cardesa et al. examines experimental and limited clinical evidence that links these disturbances to bisphosphonate-associated osteonecrosis [205]. However, it does not demonstrate a consistent increase in incidence or severity attributable to vitamin D deficiency in patients with obesity. A separate systematic review investigated oral lesions, dysgeusia, and osteonecrosis associated with COVID-19. The osteonecrosis literature primarily concerned rhinomaxillary mucormycosis, often found in patients with diabetes or corticosteroid exposure, and many of the contributing studies had methodological limitations [206]. This review offers additional context related to infection and treatment wherein oral necrosis may manifest; it should be differentiated from medication-related osteonecrosis, radiotherapy-induced injury, and proposed obesity-related marrow alterations. The review does not establish that adiposity independently causes osteonecrosis.

An additional hypothesis concerns additive effects of pre-existing metabolic hypoxia and fatty marrow changes on radiotherapy-induced vascular injury and impaired bone turnover. This proposed contribution to osteoradionecrosis warrants evaluation separately from evidence related to bisphosphonates. The clinical prevention and management of jaw osteoradionecrosis should be interpreted in accordance with the radiotherapy-specific ISOO-MASCC-ASCO guideline [207]. The assessment follows its distinct considerations of medication exposure and clinical context, as outlined in the AAOMS position paper [208]. These guidance documents support patient care within their respective indications; however, they do not endorse an additional marrow algorithm specific to obesity. Obesity remains a potential associated factor in particular treated populations, and clinical evaluation should integrate medication or radiotherapy exposure, underlying disease, local oral findings, relevant metabolic conditions, and other established risk factors.

### 6.5. Clinical and Surgical Implications

The clinical implications pertain to bone quantity, structural integrity, marrow composition, vascular support, remodeling, and healing capacity. Bone quantity and bone quality should be considered independently, as cortical thickness or apparent bone mass alone do not determine favorable microarchitecture, vascularity, remodeling activity, or regenerative potential. Variations in marrow composition and vascular support may be pertinent to bone characteristics observed during augmentation and implant procedures, but their association with obesity and their predictive significance for surgical or implant outcomes necessitate direct evaluation in humans.

Figure 3 shows an illustrative intraoperative observation of significant adipose tissue within the bone marrow identified during the process of bone harvesting for augmentation. Figure 4 demonstrates fatty degeneration, characterized by a yellow discoloration on the vestibular side of the jaw. These intraoperative findings offer descriptive clinical context regarding variations in marrow and bone appearance. The gross appearance alone does not establish histologically that the marrow is adipose, avascular, or osteonecrotic, nor does it determine whether the observed tissue alteration is attributable to obesity or other causes.

The implications of altered marrow composition and vascular support for graft integration, healing associated with implants, and postoperative recovery necessitate direct clinical assessment. Observations of marrow appearance, marginal bone changes, or hypothesized metabolic mechanisms alone are insufficient to infer primary osseointegration or implant failure. Dominiak et al. evaluated marrow steatosis in patients undergoing personalized alveolar reconstruction, offering human clinical insights pertinent to local bone characteristics without isolating a specific effect of obesity on implant failure [209]. Proper tissue handling, vascular preservation, and tension-free closure are standard surgical considerations; the technical review of flap advancement provides pertinent procedural background [210].

Clinical planning may incorporate the oral diagnosis, documented metabolic and nutritional conditions, local bone characteristics, and exposure to medications or radiotherapy. The clinical photographs in Figure 2, Figure 3 and Figure 4 are descriptive and do not independently determine graft selection, surgical technique, loading, or healing intervals. The authors nevertheless maintain that documented marrow and metabolic characteristics may help contextualize individualized planning. This proposal is informed by human reconstructive observations [209] and experimental healing findings [21], but comparative human studies are needed to determine whether phenotype-based modifications improve outcomes. Ordinary procedural care and the proposed obesity-specific extension therefore have different evidentiary bases.

## 7. Impaired Oral Wound Healing in Obesity

The assessment of oral wound healing requires the differentiation of adiposity from associated metabolic conditions, particularly insulin resistance, dysglycemia, and diabetes mellitus. When the fundamental data permit, evidence should be interpreted distinctly for cases involving obesity without documented insulin resistance or diabetes, obesity with insulin resistance or dysglycemia, and confirmed diabetes mellitus. Findings from diabetic populations or experimental models do not establish an independent effect of obesity. Similarly, studies on obesity that do not specify insulin resistance or glycemic status should not be assumed to solely represent obesity. Shared inflammatory, vascular, and metabolic pathways are discussed comprehensively in Section 3. Consequently, evidence regarding oral mucosal repair in diabetes [211] supports the interpretation that wound-healing abnormalities are associated with diabetes; however, such findings should not be generalized to obesity cases without diabetes. Hyperglycemia, advanced glycation, and other disturbances specific to diabetes constitute additional exposures that cannot be solely attributed to adiposity. The subsequent findings are evaluated based on the tissue examined, the experimental model employed, the metabolic phenotype, and the healing outcome. Therefore, evidence from studies on diabetes should be regarded as pertaining to diabetes unless the study design explicitly evaluates adiposity separately. Conversely, evidence from obesity studies should not be assumed to encompass diabetic mechanisms unless diabetes or dysglycemia was specifically present and characterized.

An experimental study on tooth extraction reported delayed alveolar healing associated with weight gain, including decreased bone fill, an increased number of TRAP-positive osteoclasts, reduced osteoprotegerin expression without a corresponding change in RANKL, and a delayed VEGF response [21]. These findings suggest potential explanations for altered socket healing. Human healing findings need to be interpreted in the context of metabolic exposure. A prospective controlled observational study compared extraction-socket healing in 56 patients with insulin-dependent diabetes and 49 controls. Delayed healing occurred in 12.5% and 8.2%, respectively, with no statistically significant between-group difference [212]. This is direct human oral evidence concerning diabetes, including a nonsignificant result, rather than proof of impaired healing attributable to obesity. It supports considering the clinical course and documented metabolic status while keeping the experimental obesity model distinct.

Findings related to leptin emphasize the necessity of differentiating between tissue expression and experimental administration. Umeki et al. identified leptin receptors within oral epithelial and vascular endothelial tissues and documented accelerated healing following topical leptin application in a rabbit oral-wound model. Their experimental cell studies demonstrated improved epithelial migration [213]. Additionally, another investigation assessed leptin expression during oral wound healing in diabetic and control rats [214]. These studies pose distinct research questions and do not substantiate the hypothesis that endogenous leptin resistance accounts for delayed oral healing in patients with obesity.

Alterations in the function of mesenchymal stromal cells provide an alternative proposed explanation. The extensive literature on wound healing delineates inflammatory modifications and diminished regenerative capacity in cells obtained from bone marrow and adipose tissue, alongside vascular and endocrine limitations on the repair process [215,216]. The specific role of these cells in oral healing, independent of diabetes and other comorbidities, warrants direct investigation.

High-energy food consumption may occur concurrently with insufficient intake or availability of nutrients essential for tissue repair [217]. Proteins, vitamins A, C, D, and E, zinc, and iron play vital roles in matrix formation, immune function, and wound healing [25]. Consequently, nutritional status warrants independent assessment from body mass index, especially when significant weight loss or bariatric surgery has affected dietary intake or absorption.

Perioperative assessment should encompass the documented metabolic and nutritional profile alongside the procedure and local oral findings. Evaluation of diabetes or dysglycemia, as well as coordination, should adhere to accepted medical criteria and the periodontal–diabetes consensus [218,219]. Insulin resistance should be recorded separately when assessed and not inferred from BMI. A nutritional evaluation may be deemed appropriate when dietary intake, significant weight loss, malabsorption, bariatric surgery, or other clinical findings suggest risk; bariatric micronutrient assessment and supplementation should follow applicable nutritional guidelines [220]. Routine testing of 25(OH)D solely due to obesity in an otherwise healthy adult lacks support from the Endocrine Society prevention guideline; this does not replace the assessment and treatment of clinically suspected or established deficiency within its own context [221]. These established considerations can coexist with the authors’ proposed utilization of metabolic phenotype to contextualize healing vulnerability, while an obesity-specific wound-healing protocol remains unvalidated.

Vitamin D plays a role in calcium regulation and the function of bone cells [222,223], and experimental research has explored its influence on osteogenic differentiation [224]. These biological insights offer a basis for examining nutritional status in the context of oral regeneration. However, they do not define an obesity-specific testing threshold nor do they prove that routine supplementation enhances outcomes for implants or surgical procedures.

Adjacent oral-healing research provides examples of strategies that could be examined within defined metabolic phenotypes. In a randomized trial involving 56 ASA I patients undergoing impacted mandibular third-molar extraction, injectable platelet-rich fibrin was shown to reduce swelling on days 3 and 7, decrease pain on days 1 and 3, and enhance wound-healing scores across assessments from days 3 to 21 [225]. A systematic review concerning autologous platelet concentrates in endodontic surgery included 14 controlled studies and reported findings related to symptoms and clinical or radiographic healing, though with heterogeneity in procedures and outcomes [226]. Neither source examined the correction of an obesity-associated healing deficit. These findings support the exploration of procedure-specific regenerative approaches; however, any application to patients with obesity necessitates direct evaluation.

Cannabinoid strategies represent a significant pathway for future research. A comprehensive systematic review conducted by Campana et al. examined the effects of cannabinoids on orofacial pain, oral inflammation, and bone healing across both clinical and preclinical studies. Variations were observed in agents, models, and outcomes, with the authors identifying a high risk of bias in the included animal studies [227]. These findings establish an experimental foundation for exploring mechanisms related to pain, inflammation, and regeneration; nonetheless, they do not establish a definitive cannabinoid treatment protocol for obesity-associated oral diseases. Such strategies remain as research proposals and ought to be evaluated in relation to specific oral health outcomes and relevant metabolic exposures.

Figure 5 illustrates tissue acquired during palatal connective tissue harvesting. The macroscopic appearance alone is insufficient to determine ectopic lipid accumulation associated with insulin resistance or to predict graft revascularization. The correlation between metabolic phenotype, palatal donor tissue composition, and graft healing has not been conclusively demonstrated by the evidence reviewed in this study.

Clinical implications should be based on the individual’s metabolic status, nutritional circumstances, the specific procedure undertaken, and the observed progression of healing. Section 9 delineates the difference between well-established clinical considerations and proposed extensions based on mechanistic evidence.

## 8. Dental Context of Obesity Pharmacotherapy

Pharmacotherapy constitutes a vital component within the comprehensive management of obesity [228]. The current guidelines for obesity treatment advocate for individualized decision-making processes, which take into account factors such as adiposity, obesity-associated complications, treatment history and responsiveness, contraindications, patient preferences, therapeutic objectives, accessibility, and regulatory indications, rather than relying on a single BMI threshold. The 2025 update to the clinical practice guideline offers the predominant contemporary framework for pharmacological obesity management [229]. Additionally, the guideline issued by the American Gastroenterological Association provides supplementary guidance regarding pharmacotherapy for adults experiencing obesity or overweight conditions accompanied by weight-related complications [230]. This dental narrative review does not establish independent eligibility thresholds for the initiation of obesity pharmacotherapy. From a dental standpoint, this matter is notably significant for two reasons: first, weight reduction alters the systemic inflammatory profile linking obesity and periodontitis; second, anti-obesity drugs, particularly GLP-1 analogs, exert both direct and indirect effects in the oral cavity, which dental professionals must consider when devising treatment plans.

The efficacy of existing treatment alternatives differs substantially. Orlistat, a lipase inhibitor for the gastrointestinal tract, resulted in an average weight reduction of 5.8 kg in the four-year XENDOS trial, in contrast to 3.0 kg in the placebo cohort [231]. The amalgamation of naltrexone and bupropion in the COR-I trial resulted in a 6.1% reduction in weight, in contrast to a 1.3% decrease in the control cohort [232]. A significant advancement was achieved with the advent of GLP-1 analogues: liraglutide at a dosage of 3.0 mg in the SCALE program resulted in an average weight reduction of 8.4 kg [233], while semaglutide 2.4 mg subcutaneously in the STEP-1 trial led to a 14.9% decrease in body weight after 68 weeks, with 86% of participants attaining a minimum of 5% loss [234]. The dual GIP and GLP-1 receptor agonist, tirzepatide, demonstrated a 20.9% reduction in weight at a 15 mg dosage over 72 weeks in the SURMOUNT-1 study, comparable to the outcomes of several bariatric surgeries [235]. Nonetheless, it is essential to recognize that obesity is a persistent and recurring condition: cessation of semaglutide treatment in the STEP-4 trial led to consistent weight reaccumulation, which also reflects the sustainability of possible periodontal advantages [236]. This highlights the importance of chronic management; whether sustained pharmacological weight reduction can induce or preserve periodontal improvements remains an independent hypothesis, necessitating oral-outcome studies.

The proposed periodontal effects of obesity pharmacotherapy require interpretation in light of drug exposure, study population, metabolic phenotype, and oral outcome. GLP-1 receptor agonists have demonstrated anti-inflammatory, osteoprotective, osteogenic, and antiresorptive effects in experimental periodontal and peri-implant models, including modulation of NF-κB and Wnt/β-catenin signaling and attenuation of alveolar bone resorption. These findings establish biological plausibility but not periodontal efficacy in humans. Available human evidence remains limited and includes observational findings, particularly in patients with type 2 diabetes; randomized clinical evidence demonstrating improvement in periodontal or peri-implant clinical outcomes remains insufficient. Interpretation therefore requires separating direct drug effects from concurrent changes in glycemic control, body weight, systemic metabolic status, dietary intake, and other treatment-related factors [237,238]. Periodontal infection may also interact with pathways related to incretins. *Porphyromonas gingivalis* has been reported to demonstrate enzymatic activity similar to DPP-4, which can degrade GLP-1, thereby suggesting a potential mechanistic connection between periodontal infection and incretin signaling. However, this observation does not establish a clinically significant reciprocal effect between periodontitis and GLP-1 receptor agonist therapy [237,238].

Potential oral adverse effects necessitate separate assessment. Dry mouth has been recognized as a pharmacovigilance signal in analyses of semaglutide. Disproportionality signals suggest disproportionate reporting; however, they do not establish incidence, absolute risk, relative risk, or causality. It has been proposed that altered GLP-1 receptor signaling in salivary glands, including prolonged receptor activation and β-arrestin-mediated desensitization, may serve as a mechanism for decreased salivary secretion. This mechanistic evidence supports biological plausibility but does not confirm a causal effect of the drug [239,240]. Reduced salivary secretion may impair oral clearance, buffering capacity, and remineralization, thereby heightening susceptibility to caries, dentin hypersensitivity, candidiasis, halitosis, and oral discomfort. These outcomes are biologically consistent with salivary hypo-function; however, their incidence and causal relationship with GLP-1 receptor agonists remain insufficiently defined [240]. Gastrointestinal adverse effects, including nausea, vomiting, and gastroesophageal reflux, may also increase dental tissue exposure to gastric contents, contributing to enamel erosion. The extent of this risk depends on the frequency and duration of gastrointestinal symptoms and should not be inferred solely from medication exposure [240,241].

Postoperative healing necessitates individual consideration. Although healing complications following implant surgery and dental extraction have been documented, a causal relationship with GLP-1 receptor agonist exposure has not been established. Rapid and significant weight loss may also be associated with decreased dietary intake, protein deficiency, or micronutrient deficiency, which can potentially impact the condition of the oral mucosa and the healing of soft tissues and bones. Such effects should be distinguished from the direct pharmacological actions of the medication [241].

A dental assessment may encompass the evaluation of current anti-obesity pharmacotherapy, duration of treatment, recent dose escalation, gastrointestinal adverse effects, xerostomia or other salivary symptoms, and relevant nutritional modifications. Clinically significant xerostomia may necessitate hydration, saliva substitutes, topical fluoride applications, and additional preventive follow-up tailored to salivary function and caries susceptibility. Delayed gastric emptying introduces a separate risk of aspiration during sedation or general anesthesia. The multi-society guidelines identify dose escalation, elevated dosages, gastrointestinal symptoms, and other related conditions associated with delayed gastric emptying as pertinent risk factors [242]. A review of dental anesthesia and a correspondence in the British Dental Journal discuss the implications for dental procedures requiring sedation; these should be regarded as professional interpretations rather than independent multisociety directives [243,244]. The scheduling of an elective procedure beyond the period of rapid dose escalation may be considered when sedation, general anesthesia, symptoms, or other aspiration risk factors are present, in coordination with the anesthesia and prescribing teams. This does not establish a universal timing requirement for invasive dental procedures performed under local anesthesia alone. The recommendations from Australian and New Zealand multisociety guidelines in 2025 offer an additional jurisdiction-specific approach; guidance varies regarding medication continuation, preparation, and postponement, and therefore management should adhere to applicable guidance and individual risk assessment [245].

## 9. Translational Implications for Chairside Assessment and Management

Clinical interpretation distinguishes established care, human observations, and emerging proposals utilizing the categories A, B, and C as defined in Section 2.3. CBC-derived indices, including SII, SIRI, and AISI, are examined as potential indicators of systemic inflammatory status [62]. The referenced editorial endorses their investigation, rather than definitive clinical validation of dental thresholds. Human peri-implant research and their syntheses report associations with selected systemic inflammatory and lipid measurements [161,246]. These findings do not specify the anatomical origin of circulating signals nor validate every composite index. We maintain inflammatory burden as contextual information when independently accessible; however, proposed diagnostic, prognostic, and monitoring applications necessitate demonstration of incremental value beyond periodontal and peri-implant examinations and relevant medical information.

Salivary adipokines, endocrine context, and body composition similarly merit further investigation. Human salivary and gingival findings support associated measurements [52,53,54,104], while endocrine studies suggest that factors such as sex, hormonal exposure, and metabolic context can influence interpretation [57,58]. Salivary panels and integrated metabolic profiles are still proposed tools, with their reproducibility, thresholds, predictive performance, and clinical utility requiring thorough evaluation. Standard risk-based diabetes assessment and referral procedures remain applicable when medical indications are present [218,247]. A proposed phenotype framework does not replace those established indications.

Potential research strategies include adipokine signaling, microRNA regulation, macrophage-related epigenetic pathways, microbiome-directed approaches, and regenerative interventions. Observations in human tissues and experimental studies provide distinct support for these ideas [92,102,104,105]. Probiotics, dietary modification, and periodontal therapy may be investigated for their separate effects on oral disease and systemic metabolic measurements [248,249]. The authors’ hypotheses remain part of the review even when clinical validation is incomplete. Interventional studies, including those before and after substantial weight loss, should distinguish adiposity-related changes from concurrent differences in glycemic control, nutrition, medication, and oral care.

Periodontal and peri-implant maintenance adheres to diagnosis, treatment response, and established risk factors in accordance with the applicable EFP guidance [250,251]. The authors suggest that a documented metabolically adverse phenotype may provide additional context when considering the appropriateness of closer observation in patients with active disease or other risk factors. This extension is classified as Category C, based on observations categorized as B [8,161], and does not establish a universal obesity-specific interval of four to six weeks. Implant loading is similarly contingent upon the procedure, primary stability, augmentation, local and prosthetic factors, and host condition; the cited ITI consensus specifically pertains to single implants in the anterior maxilla [252]. While the general obesity–implant relationship offers contextual observational insight [20], no specific loading threshold based solely on obesity can be derived from these references.

Table 1 delineates mechanisms, clinical findings, and potential interventions. Table 2, Table 3 and Table 4 systematize surgical considerations, the proposed phenotype taxonomy, and dental findings that may necessitate medical assessment. Each source is designated to the corresponding action or interpretation it endorses. Established A considerations continue to be relevant within their current indications; B observations remain clinically valuable; C components incorporate emerging biomarkers, strategies, and authors’ proposals. The comprehensive framework is conceptual and has not been validated as a prediction model, diagnostic system, or standalone treatment protocol.

**Table 1:** Mechanism-to-clinic crosswalk.

Mechanism/Phenotype	Clinical Proxy	Oral Relevance	Chairside Implication	Evidence Basis/Clinical Readiness
Insulin resistance or dysglycemia.	HbA1c and fasting plasma glucose when indicated; HOMA-IR or HOMA2-IR when measured for research or metabolic characterization, with the method specified.	Periodontitis association; healing relevance is stronger for diabetes; primary osseointegration is not established.	Coordinate documented diabetes and dysglycemia; test or refer according to accepted medical criteria. Consider documented insulin resistance as contextual information when interpreting periodontal findings. HOMA2-IR remains an emerging research measure and does not define a dental diagnostic, loading, or surgical rule.	A—[247]: indicated diabetes testing; [218]: periodontal–diabetes coordination. B—[150]: cross-sectional periodontal association using HOMA-IR, not HOMA2-IR. C—[253]: HOMA methodology; supports specifying HOMA2, not dental validation. The integrated phenotype interpretation is the authors’ proposal.
Chronic low-grade inflammation.	CRP; IL-6; TNF-α; SII, SIRI, and AISI.	Systemic inflammatory associations have been reported; CBC indices are not validated local-disease surrogates and should be assessed in the context of systemic low-grade inflammation associated with obesity.	Use periodontal and peri-implant examinations and relevant medical history to guide decisions. When available, systemic inflammatory burden may inform the clinical context. Composite CBC indices remain research candidates and should not serve as stand-alone rules for local disease or treatment.	A—[250,251]: established periodontal and peri-implant assessment and care. B—[161]: human peri-implant inflammatory and lipid associations. C—[254]: editorial rationale for CBC-derived indices; no validated dental thresholds.
Oral dysbiosis and oral–gut axis.	Clinical periodontal status; microbiome profiling.	Human associations and microbial transmission findings are supported by experimental and mechanistic evidence. The Najeeb study has conflicting prevalence estimates and sequencing descriptions; its microbiological findings require particular caution. An obesity-specific causal oral–gut pathway remains unvalidated.	The oral–gut construct may guide hypothesis development and interpretation. Provide indicated periodontal and dietary care. Microbiome profiling remains a research approach rather than a routine tool for selecting obesity-specific therapy.	A—[251]: established periodontal care; [109]: WHO-oriented free-sugar guidance. B—[91]: human oral-to-gut transmission; [95]: cross-sectional associations with serious reporting limitations. C—[14,92]: proposed pathways and intestinal-disease context. The obesity-specific integration is an authors’ hypothesis.
Epigenetic and adipokine pathways.	No validated chairside marker.	Exploratory human gingival tissue evidence and experimental cell and animal evidence are available; however, no validated treatment-selection thresholds exist.	Use these pathways as mechanistic and hypothesis-generating context. Routine adipokine or epigenetic panels still lack validated treatment-selection thresholds.	B—[104]: measured human gingival microRNA expression. C—[104]: predicted targets and proposed clinical interpretation; [105]: experimental JMJD3, adiponectin, and macrophage pathways.
Micronutrient and vitamin D deficiency.	25(OH)D or other tests when clinically indicated.	Deficiency may affect bone and repair; routine obesity-based presurgical screening for 25(OH)D is not supported.	Target testing to an established indication, suspected or documented deficiency, malabsorption, bariatric nutritional risk, or other clinical suspicion. Treat or coordinate confirmed vitamin D deficiency according to appropriate guidance.	A—[221]: guidance against routine 25(OH)D testing in otherwise healthy adults with obesity; this prevention guideline does not supply a universal deficiency-treatment protocol. A—[220]: assessment and supplementation for applicable bariatric micronutrient indications.
Marrow and therapy-related bone risk.	Local imaging and clinical findings; history of antiresorptive and antiangiogenic therapy or radiotherapy.	Human jaw lesions and reconstructive observations document marrow and local bone variation. The obesity-specific marrow phenotype remains unvalidated. MRONJ and osteoradionecrosis have distinct medication and radiotherapy contexts.	Apply medication-specific MRONJ assessment and radiotherapy-specific osteoradionecrosis guidance. Documented marrow composition and local bone-quality findings may inform the clinical context. The proposed obesity-related marrow phenotype remains a research construct; obesity or gross appearance alone does not diagnose osteonecrosis or predict implant failure.	A—[208]: MRONJ guidance; [207]: osteoradionecrosis guidance. B—[209]: human reconstructive observations; [17]: synthesis of descriptive jaw-lesion observations, with nonstandardized terminology. C—[205]: proposed vitamin D and bisphosphonate interactions; the obesity-specific marrow framework is an authors’ proposal.
Oxidative stress and dyslipidemia.	Lipid profile when medically indicated; oxidative markers.	Oxidative and lipid associations have been reported; however, no validated threshold for dental biomarkers exists.	Manage known dyslipidemia. Consider known dyslipidemia and oxidative burden as potential contextual modifiers of oral inflammatory risk; do not initiate lipid or oxidative-marker testing solely for dental risk without an independent medical indication.	B—[71]: human oxidative-stress measurements; [161]: human lipid and peri-implant associations. C—The proposed use of those findings for an integrated dental phenotype or a biomarker threshold is an authors’ extension. Neither previous paper validates testing solely to predict dental outcomes.
Wound-healing vulnerability.	Clinical healing; glycemic and nutritional status.	Obesity-specific oral-healing evidence is predominantly experimental. In a human diabetic extraction study, delayed healing occurred in 12.5% of patients with insulin-dependent diabetes and 8.2% of controls, without a significant difference between groups. Nutritional evidence supports assessment within clinical indications.	Use procedure-appropriate surgical care. First, evaluate local causes of abnormal healing, then consider targeted glycemic and nutritional assessment when indicated. A documented metabolic phenotype may contextualize healing vulnerability; this does not constitute a validated obesity-specific surgical rule.	A—[220]: indicated bariatric nutritional care; [247]: indicated glycemic assessment. B—[212]: prospective human extraction study with a nonsignificant comparison. C—[212]: rat extraction-healing experiment; phenotype-specific extrapolation is proposed.

Evidence codes: A, established care within the applicable indication and source scope; B, reasonable considerations supported by human clinical observations or their syntheses; C, mechanistic evidence, expert interpretation, emerging strategies, or authors’ proposals. Codes apply to the specified component and are not a formal GRADE certainty rating. A B-supported observation may inform a C framework. C does not signify that a proposal should be discarded. A source can have different roles for different claims. DOI assignments specify the supported component. Abbreviations: AISI, Aggregate Index of Systemic Inflammation; CBC, complete blood count; CRP, C-reactive protein; HbA1c, glycated hemoglobin A1c; HOMA-IR, homeostatic model assessment of insulin resistance; HOMA2-IR, homeostatic model assessment 2 of insulin resistance; IL-6, interleukin-6; MRONJ, medication-related osteonecrosis of the jaw; SII, Systemic Immune-Inflammation Index; SIRI, Systemic Inflammatory Response Index; TNF-α, tumor necrosis factor alpha; 25(OH)D, 25-hydroxyvitamin D.

**Table 2:** Obesity phenotype-aware surgical planning checklist.

Domain	Key Considerations	Clinical Implications	Evidence Basis /Clinical Readiness
Preoperative Assessment	Metabolic phenotype	Record obesity, central adiposity, MetS, dysglycemia, diabetes, dyslipidemia, and treatment exposures separately. Consider the documented metabolic phenotype when interpreting potential healing and inflammatory vulnerability. This phenotype-aware integration is an authors’ proposed clinical consideration, not a validated perioperative algorithm.	B—[8]: obesity–periodontitis synthesis; [255]: metabolic syndrome components and periodontitis; [150]: cross-sectional insulin-resistance mediation model. C—The integrated perioperative phenotype assessment is an authors’ proposal informed by these B findings.
	Glycemic status	Coordinate known diabetes and dysglycemia; perform HbA1c or FPG testing, or refer when accepted screening or clinical criteria are met. Observational periodontal associations may increase clinical awareness, but testing should remain indication-based.	A—[247]: diabetes screening and diagnostic criteria; [218]: periodontal–diabetes assessment and coordination within their indications.
	Vitamin D and micronutrients	Avoid routine obesity-only 25(OH)D screening. Assess established indications, deficiency risk, malabsorption, bariatric nutritional history, and other clinical findings. Documented deficiency may be relevant to bone and repair biology, while its incremental value in an obesity-specific surgical phenotype remains debated.	A—[221]: prevention and testing scope; [220]: bariatric micronutrient assessment and supplementation. C—The additional phenotype-specific surgical interpretation is an authors’ proposal; these guidelines do not validate it.
	Dyslipidemia and inflammatory burden	Document known disease. Consider known dyslipidemia or systemic inflammatory burden as contextual phenotype information when independently available; do not order a CRP or lipid panel solely to predict peri-implant outcomes.	B —[161]: human peri-implant inflammatory and lipid associations. C —[254]: proposed CBC-index interpretation. Neither source validates routine lipid or CRP testing to predict peri-implant outcomes.
	OSA suspicion and CPAP use	Assess symptoms, history, and treatment when OSA is suspected. A risk questionnaire may help identify the need for medical or sleep-specialist assessment, but it does not diagnose OSA. The cited STOP-Bang synthesis primarily concerns sleep-clinic populations, so its performance should not be assumed for all unselected dental attendees.	A—[256]: comprehensive evaluation and diagnostic testing for suspected adult OSA; questionnaires alone are not diagnostic. B —[257]: STOP-Bang diagnostic-performance synthesis in sleep-clinic populations.
	Antiresorptive and antiangiogenic therapy	Assess exposure, indication, local oral factors, and planned surgery; coordinate according to MRONJ guidance. Obesity status does not replace treatment-exposure assessment.	A —[208]: medication exposure, underlying indication, oral factors, and procedure-specific MRONJ assessment.
Intraoperative Considerations	Surgical technique and vascular preservation.	Use procedure-appropriate tissue handling, preserve vascularity where relevant, and obtain tension-free closure when required by the procedure. A technical review describes practical flap-advancement methods. Human alveolar-reconstruction observations may inform evaluation of local bone characteristics. Additional modification according to the proposed obesity phenotype remains an authors’ consideration requiring comparative clinical evaluation.	C—[210]: expert procedural review describing flap advancement and closure; this is clinical background, not an obesity-specific guideline. B—[209]: human alveolar-reconstruction and marrow observations. C—[21]: experimental rat healing. Phenotype-driven changes are authors’ proposals; no A-level obesity-specific surgical modification is established.
Maintenance	Periodontal and peri-implant recall.	Individualize periodontal and peri-implant maintenance according to diagnosis, treatment response, and established risk factors. The authors propose that a documented metabolically adverse phenotype may warrant closer observation when active disease or other risk factors are present. The interval remains individualized; no universal obesity-specific 4–6-week recall interval is prescribed.	A—[250,251]: individualized periodontal and peri-implant maintenance. B—[8,161]: human obesity-related periodontal and systemic peri-implant associations. C—Additional phenotype-based intensity of observation is the authors’ proposed extension.
Postoperative	Delayed healing and nutritional reassessment.	First, evaluate local causes of delayed healing. Add a glycemic or nutritional assessment when the clinical course, medical history, or accepted screening criteria indicate risk. Abnormal healing is a reasonable contextual prompt but is not a specific metabolic diagnostic marker. The cited human diabetic study did not demonstrate a significant increase in delayed healing.	A—[220]: indicated bariatric nutritional assessment; [247]: indicated glycemic assessment. B—[212]: prospective human extraction observations, including a nonsignificant group difference. C—[21]: experimental healing; phenotype interpretation is proposed.

This table systematically categorizes perioperative considerations according to the stage of care. Established procedural and medical considerations are differentiated from observational human findings and the proposed modifications tailored to specific phenotypes. Each action is interpreted in relation to the procedure, the documented conditions, and the supporting source. Evidence codes: A, established care within the applicable indication and source scope; B, reasonable considerations supported by human clinical observations or their syntheses; C, mechanistic evidence, expert interpretation, emerging strategies, or authors’ proposals. Codes apply to the specified component and are not a formal GRADE certainty rating. A B-supported observation may inform a C framework. C does not signify that a proposal should be discarded. A source can have different roles for different claims. DOI assignments specify the supported component. Abbreviations: BMI, body mass index; CPAP, continuous positive airway pressure; CRP, C-reactive protein; EFP, European Federation of Periodontology; FPG, fasting plasma glucose; HbA1c, glycated hemoglobin A1c; HOMA-IR, homeostatic model assessment of insulin resistance; ITI, International Team for Implantology; MetS, metabolic syndrome; MRONJ, medication-related osteonecrosis of the jaw; OSA, obstructive sleep apnea; STOP-Bang, Snoring, Tiredness, Observed apnea, high blood Pressure, Body mass index, Age, Neck circumference, and Gender; 25(OH)D, 25-hydroxyvitamin D.

**Table 3:** Proposed hypothesis-generating obesity phenotype taxonomy framework for dental risk.

Phenotype	Dominant Context	Oral Evidence	Clinical Implication	Evidence Basis/Clinical Readiness
Central adiposity and MetS.	Inflammation, dyslipidemia, endothelial dysfunction, and metabolic dysfunction.	Human observational evidence concerns periodontitis and individual components of metabolic syndrome. The cited peri-implant review found no study evaluating metabolic syndrome as a complete entity; it does not validate a unified peri-implant metabolic syndrome category.	Use established dental assessment and record central adiposity and diagnosed metabolic syndrome separately. Documented components may inform periodontal and peri-implant interpretation and coordinated management. The integrated phenotype taxonomy is an authors’ proposal and is not a validated risk algorithm.	B—[255]: metabolic syndrome components and periodontitis; [258]: peri-implant findings for individual components, with no complete-syndrome study. C—Integrated phenotype taxonomy and proposed management interpretation.
Obesity with insulin resistance or dysglycemia.	Hyperinsulinemia, impaired glucose metabolism, and diabetes-specific AGE pathways when diabetes is present.	Periodontal association is supported; healing concerns are stronger for diabetes; primary osseointegration impairment attributable to obesity remains unproven.	Coordinate documented diabetes or dysglycemia under established indications. Documented insulin resistance or dysglycemia may inform interpretation of periodontal findings. Distinguish healing evidence from diabetes from insulin resistance without diabetes. No obesity-only loading or surgery rule follows.	A—[218]: periodontal–diabetes coordination. B—[150]: human cross-sectional HOMA-IR associations. C—[211]: diabetes-related oral repair mechanisms; extension to insulin resistance without diabetes or to an obesity-only surgical rule remains proposed.
Obesity with documented vitamin D deficiency.	Impaired mineral metabolism when deficiency is confirmed.	The potential bone and healing relevance is biologically plausible; however, an independent obesity-specific effect on MRONJ or implant risk remains unestablished.	Treat or coordinate the confirmed deficiency. Consider documented deficiency as a potential modifier of bone and healing biology; do not screen all adults with obesity solely on phenotype.	A—[221]: against routine obesity-only testing in its prevention scope; [220]: assessment and supplementation within applicable bariatric indications. C—[205]: vitamin D and bisphosphonate-related jaw mechanisms. These sources do not establish an independent obesity-specific MRONJ risk.
Early post-bariatric state.	Rapid dietary and metabolic change; possible micronutrient deficiency.	Heterogeneous periodontal and oral changes have been reported; no fixed implant-risk window has been established.	Document the procedure, intake, supplementation, and current metabolic and nutritional stability. Consider the early post-bariatric state as a distinct clinical context for individualized prevention and elective treatment planning without imposing an unvalidated fixed delay.	B—[156,157]: clinical postoperative periodontal observations; [158]: evidence synthesis. C—The integrated early postoperative phenotype and proposed timing interpretation are authors’ considerations. No fixed implant-risk window follows.
Late post-bariatric state.	Metabolic improvement may coexist with persistent micronutrient deficiency.	Periodontal outcomes remain heterogeneous; elapsed time does not establish normalization.	Reassess current metabolic and nutritional status rather than assuming recovery. Retain post-bariatric history as a clinically relevant phenotype descriptor when persistent nutritional or oral risk is present.	A—[220]: ongoing bariatric nutritional care. B—[153]: original clinical one-year follow-up; [154]: periodontal evidence synthesis. C—The late postoperative phenotype is a proposed contextual category; elapsed time does not validate recovery.
High systemic inflammatory burden and high CRP.	Nonspecific systemic inflammatory phenotype.	Associations exist between systemic inflammatory burden and periodontal or peri-implant inflammation; however, no validated dental CRP threshold has been established.	When CRP is clinically available, consider elevated systemic inflammatory burden as context for phenotype-aware risk interpretation. Do not use CRP as a stand-alone dental diagnostic or treatment threshold.	B —[259]: cross-sectional CRP–periodontitis findings in a population with obesity; [246]: peri-implantitis and systemic inflammation synthesis, not an obesity-specific phenotype study. C—Clinical interpretation of a high-CRP phenotype and any dental threshold remain proposed.
Dyslipidemic phenotype.	Atherogenic lipids with a metabolic and inflammatory co-phenotype.	Cross-sectional associations between dyslipidemia and peri-implant inflammatory findings are reported.	Consider known dyslipidemia a potential systemic modifier when interpreting peri-implant inflammatory findings. Lipid testing initiated solely because of peri-implant disease remains unvalidated.	B—[161]: human cross-sectional lipid and peri-implant inflammatory associations. C—The proposed phenotype interpretation and lipid screening solely to predict peri-implant outcomes are not validated by that study.
Obesity without documented metabolic abnormality.	Adiposity without confirmed dysglycemia, dyslipidemia, or deficiency.	General obesity–periodontitis and implant associations provide context, but the cited reviews do not separately validate a metabolically healthy obesity category. The absence of a documented abnormality does not prove metabolic normality.	Use standard dental risk assessment. Retain obesity as contextual information in phenotype-aware interpretation, but do not escalate laboratory testing or surgical treatment based solely on BMI without corroborating metabolic or clinical risk.	B—[8,20]: general obesity-related oral associations. C—Obesity without a documented metabolic abnormality is an author’s proposed interpretive category, not a validated dental risk class.

This table delineates an emerging phenotypic framework for dental interpretation. The categories may exhibit overlap and have not been validated as discrete diagnostic or prognostic classes. Established care practices remain pertinent within their specified indications, whereas human observations and proposed extensions are identified independently. Evidence codes: A, established care within the applicable indication and source scope; B, reasonable considerations supported by human clinical observations or their syntheses; C, mechanistic evidence, expert interpretation, emerging strategies, or authors’ proposals. Codes apply to the specified component and are not a formal GRADE certainty rating. A B-supported observation may inform a C framework. C does not signify that a proposal should be discarded. A source can have different roles for different claims. DOI assignments specify the supported component. Abbreviations: AGE, advanced glycation end-product; BMI, body mass index; CRP, C-reactive protein; HOMA-IR, homeostatic model assessment of insulin resistance; MetS, metabolic syndrome; MRONJ, medication-related osteonecrosis of the jaw. The phenotype categories are authors’ proposed contextual descriptions and may overlap; they are not validated diagnostic or prognostic classes.

**Table 4:** Dental findings that may prompt medical risk assessment or referral.

Dental Presentation	Possible Systemic Issue	Recommended Action	Evidence Basis/Clinical Readiness
Periodontitis with diabetes risk factors or symptoms.	Prediabetes or type 2 diabetes.	Use a risk-based HbA1c or FPG assessment, or a medical referral; periodontitis alone is not diagnostic. The periodontal presentation may increase awareness of diabetes risk, but testing should still follow accepted medical criteria and the broader clinical context.	A—[247]: risk-based diabetes assessment; [218]: periodontal–diabetes assessment and medical coordination.
Unexpectedly severe or refractory periodontitis after assessing local causes.	Possible systemic modifier.	Review medical history and established systemic risk factors. Unexpected severity may justify considering a systemic modifier as an authors’ reasonable clinical consideration; perform targeted dysglycemia evaluation only when accepted screening criteria or clinical suspicion are present.	A—[218,247]: testing and referral within accepted indications. B—[150]: cross-sectional periodontal and metabolic association. C—Unexpected severity as an additional contextual prompt is an authors’ proposal, not a validated screening rule.
Severe peri-implant inflammation with limited local explanation.	Possible dyslipidemic or systemic inflammatory co-phenotype.	Evaluate local causes first. Consider known dyslipidemia or systemic inflammatory burden as potential contextual modifiers. Perform lipid testing only if broader medical risk independently supports it; peri-implant inflammation alone is not a validated screening trigger.	B—[161]: human systemic lipid and peri-implant inflammatory associations. C—The proposed contextual interpretation does not establish severe peri-implant inflammation as an independent lipid-screening trigger.
Recurrent or unexplained marginal bone loss.	Possible mineral or bone disorder only with corroborating evidence.	Evaluate local and prosthetic causes first. Consider mineral metabolism or vitamin D deficiency in the differential when history, symptoms, malabsorption, bariatric status, bone disease, or other evidence creates clinical suspicion; perform 25(OH)D or mineral testing only when an established indication or clinical suspicion exists.	A—[250]: peri-implant assessment; [221]: vitamin D testing scope; [220]: applicable bariatric deficiency assessment. C—[205]: vitamin D and jaw mechanisms. The proposed mineral differential is contextual; no obesity-specific marginal-bone-loss algorithm is validated.
Snoring, witnessed apnea, sleepiness, morning dry mouth, and other OSA risk features.	Obstructive sleep apnea.	Review the overall OSA history, symptoms, and risk profile. A questionnaire can help identify the need for medical or sleep-specialist evaluation but does not diagnose OSA. The cited STOP-Bang performance estimates apply mainly to sleep-clinic populations and should not be assumed to apply to all dental attendees.	A—[256]: comprehensive evaluation and diagnostic testing. B—[257]: STOP-Bang performance in sleep-clinic populations.
Sleep bruxism with OSA features.	Sleep-disordered breathing.	Consider sleep assessment when bruxism coexists with a suggestive OSA risk profile; refer only when the overall OSA risk is suggestive because bruxism alone is nonspecific.	B—[196]: sleep bruxism and OSA observations. A—[256]: appropriate OSA evaluation when the overall clinical presentation supports it; isolated bruxism is not diagnostic.
Delayed extraction or surgical healing.	Dysglycemia, diabetes, medication effect, nutritional risk, or vascular factor.	Assess local causes first. Delayed healing may prompt reconsideration of documented metabolic and nutritional circumstances; undertake targeted assessment when the history, clinical course, or accepted medical criteria indicate risk. The cited diabetic extraction study showed a nonsignificant group difference, so delayed healing is not established as a specific metabolic screening sign.	A—[220]: applicable bariatric nutritional care; [247]: indicated glycemic assessment. B—[212]: human extraction healing, including the nonsignificant result. C—[21]: experimental rat healing; the integrated clinical prompt is proposed.
Antiresorptive or antiangiogenic exposure, planned surgery, or suspicious jaw findings.	MRONJ risk.	Use exposure-specific MRONJ prevention and diagnostic guidance; obesity is not a diagnostic criterion. Consider metabolic phenotype only as contextual information and do not substitute it for established medication, disease, and local risk assessment.	A—[208]: exposure-specific MRONJ prevention and diagnostic guidance. C—Any additional obesity-phenotype interpretation remains contextual and does not substitute for established exposure and oral assessment.
Halitosis with persistent gastrointestinal symptoms after addressing oral causes.	Possible extraoral gastrointestinal cause: oral–gut hypothesis.	Evaluate and treat intraoral causes first. Consider medical or gastrointestinal evaluation if persistent gastrointestinal symptoms remain, based on clinical indications. Retain the oral–gut relationship as a hypothesis-generating context, but do not diagnose gut dysbiosis from halitosis.	B—[260]: systematic review of halitosis etiologies and associations. C—[261]: narrative oral–gastrointestinal context; the referral interpretation follows persistent symptoms and ordinary clinical indications, not a validated halitosis-to-gut-dysbiosis rule.

This table organizes dental findings and accompanying clinical information that may prompt medical risk assessment or referral. The findings are nonspecific and require an appropriate differential diagnosis. Established referral indications are distinguished from authors’ proposed contextual prompts. Evidence codes: A, established care within the applicable indication and source scope; B, reasonable considerations supported by human clinical observations or their syntheses; C, mechanistic evidence, expert interpretation, emerging strategies, or authors’ proposals. Codes apply to the specified component and are not a formal GRADE certainty rating. A B-supported observation may inform a C framework. C does not signify that a proposal should be discarded. A source can have different roles for different claims. DOI assignments specify the supported component. Abbreviations: BMI, body mass index; CRP, C-reactive protein; FPG, fasting plasma glucose; HbA1c, glycated hemoglobin A1c; MRONJ, medication-related osteonecrosis of the jaw; OSA, obstructive sleep apnea; STOP-Bang, Snoring, Tiredness, Observed apnea, high blood Pressure, Body mass index, Age, Neck circumference, and Gender; 25(OH)D, 25-hydroxyvitamin D.

## 10. Strength of Evidence, Uncertainties, and Limitations of the Current Literature

The evidence base draws upon human observational studies and their syntheses, clinical interventions, professional guidance, and experimental research related to oral and systemic tissues. Periodontitis exhibits substantial observational support for an association with adiposity; however, the predominance of cross-sectional studies and less consistent evidence for independent longitudinal progression are noted [8,148,149]. Evidence concerning peri-implant conditions emphasizes inflammatory and radiographic assessments over primary osseointegration or implant survival [20,161]. Human studies on salivary, hormonal, microbial, and gingival expression identify measurable associations [34,52,58,89,104], whereas many mechanisms involving the oral–gut axis, gut–bone axis, and regulatory processes predominantly rely on animal models, in vitro experiments, or other tissue and disease contexts [92,102,103,105]. Evidence pertaining to the jawbone includes heterogeneous pediatric imaging and clinical reconstructive observations [15,16,209]; the data reported by Temur et al. do not demonstrate a correlation between lower cortical width or fractal dimension and obesity [16]. Research into oral healing combines experimental obesity models [21], diabetes-specific studies [211,214] and limited direct human comparisons, including a nonsignificant comparison of extraction healing [212]. The synthesis related to adult caries is nonsignificant [163], whereas pediatric syntheses reveal positive and uncertain findings [164,165,166].

The study design, methodological quality, directness, and certainty pertaining to a specific conclusion are consequently distinct issues. A meta-analysis encompasses the limitations inherent in its contributing studies, and overlapping reviews do not constitute independent verification. The categories A, B, and C delineate clinical readiness and source roles rather than serving as a formal grading of certainty. Observational findings and mechanistic hypotheses continue to be pertinent to clinical reasoning and research advancement; however, any assertion of stronger causal, diagnostic, prognostic, or therapeutic claims necessitates evidence commensurate with such claims.

Exposure classification represents a significant source of heterogeneity. Metrics such as BMI, waist circumference, waist-to-hip ratio, and other adiposity indicators are not interchangeable; furthermore, variations in thresholds and population characteristics impede comparability [148,262]. BMI does not provide a direct assessment of fat distribution; as a result, findings based on BMI may not be generalizable to central adiposity. Additionally, conditions such as diabetes, insulin resistance, and metabolic syndrome necessitate careful differentiation from adiposity. Their roles as confounders, mediators, or effect modifiers are contingent upon the causal framework and the temporal sequence. Factors including smoking, diet, socioeconomic status, medication use, and oral hygiene further introduce potential confounding variables. Incomplete measurement or adjustment for these factors may lead to residual confounding; statistical adjustment alone is insufficient to establish an effect specifically attributable to obesity.

Cross-sectional associations are insufficient to establish temporal order or exclude reverse causality. Consequently, proposed reciprocal relationships between periodontal inflammation and metabolic dysfunction remain distinct from confirmed causal effects in either direction [262]. Longitudinal evidence concerning oral disease progression and treatment outcomes remains limited compared to data on prevalent disease. In a ten-year cohort study, the unadjusted association between central obesity and the progression of periodontitis did not persist after multivariable adjustment [149]. Repeated measurements of adiposity, metabolic conditions, treatment exposures, and oral outcomes are necessary to elucidate these relationships.

Outcome heterogeneity further constrains synthesis. Variations in definitions of periodontitis and peri-implantitis, differences in examination protocols, and inconsistent follow-up procedures complicate comparisons. Measures such as bleeding on probing, probing depth, attachment loss, marginal bone loss, peri-implant disease, primary osseointegration, and implant survival are considered discrete outcomes [8,20]. An association with an inflammatory or radiographic parameter does not inherently imply a prediction of progressive disease, failed osseointegration, or implant loss.

Biomarker findings necessitate similarly precise interpretation. Measurements from serum, saliva, gingival crevicular fluid, and tissue correspond to distinct biological compartments; variations in sampling methods, assay techniques, and local inflammatory activity may result in inconsistent outcomes [34]. Changes observed following periodontal treatment should also be interpreted considering the comparator, concurrent interventions, and follow-up assessments. Improvements observed in inflammatory or metabolic measurements do not, in isolation, confirm a reduction in cardiovascular events, the prevention of diabetes, or indicate enduring improvements in oral health outcomes [152].

Multiple proposed mechanisms involving adipokines, epigenetics, the oral–gut axis, and the gut–bone axis are based on experimental models or evidence derived from non-oral tissues and other disease contexts [92,102,105]. Their clinical relevance is contingent upon the species, tissue type, metabolic state, exposure, and outcome examined. Findings obtained from diabetic models cannot be inherently ascribed to obesity absent of diabetes, nor can osteonecrosis observed in animals exposed to zoledronic acid and undergoing tooth extraction be used to quantify risk in patients who are untreated [204,214]. Pediatric jawbone measurements, observations from adult surgical procedures, marrow adiposity, and histologically confirmed necrosis necessitate separate interpretation. Clinical photographs serve to illustrate findings but do not establish causality, prevalence, or prognostic significance.

The literature reviewed herein does not establish an externally validated dental risk algorithm for the proposed obesity phenotypes. This limitation pertains to the integrated framework and its unvalidated extensions; it does not undermine established care practices or the human observations that inform them. In Table 1, Table 2, Table 3 and Table 4, section A delineates standard-of-care considerations within their respective indications, section B identifies reasonable considerations supported by human observations, and section C highlights emerging biomarkers, strategies, and proposals by authors. Circulating indices, salivary adipokines, and phenotype combinations necessitate assessment of reproducibility, calibration, discrimination, incremental value, and their influence on decision-making and outcomes before they can provide individual risk estimates or inform new treatment protocols. The current indications for medical testing, referrals, periodontal maintenance, and exposure-specific surgical assessments remain applicable. The proposed framework serves as a structure for further investigation and clinical contextualization, rather than as validated diagnostic criteria or an independent treatment protocol.

As a narrative synthesis encompassing a variety of exposures and outcomes, this review is susceptible to selection and interpretive bias. Its conclusions are limited by the populations, measurement methods, and follow-up durations of the studies cited. The qualitative synthesis provided should not be regarded as a definitive certainty rating for each clinical outcome.

## 11. Future Directions and Conclusions

Obesity-related oral findings are most appropriately interpreted when considered in conjunction with the measured exposure, associated metabolic conditions, and oral health outcomes. Human evidence substantiates associations with periodontitis and certain peri-implant inflammatory and radiographic measurements, whereas caries findings exhibit heterogeneity and are often compatible with shared determinants. Observations from salivary, hormonal, microbial, and tissue observations provide complementary insights. Evidence concerning jawbone and oral healing varies in directness, encompassing pediatric imaging, clinical surgical observations, diabetes-specific findings, and experimental models. Collectively, these findings underscore the importance of investigating the metabolic context within dental care, without asserting a definitive causal relationship between adiposity and oral health, nor relying solely on BMI for prognosis.

Obesity may influence certain peri-implant outcomes; however, it is not, in itself, a contraindication to implant therapy. Clinical interpretation considers the oral diagnosis, diabetes and other documented metabolic conditions, medication and radiotherapy exposure, nutritional circumstances, as well as behavioral and socioeconomic determinants. Established care continues to be based on its relevant indications. Human observational findings can reasonably inform the clinical context, whereas decisions guided by biomarkers and phenotype-specific procedural modifications remain proposed extensions. Table 1, Table 2, Table 3 and Table 4 explicitly delineate these distinctions through categories A, B, and C. This framework aims to inspire testable hypotheses and thoughtful, individualized interpretation; its integrated predictive and interventional utility remains to be validated.

Future studies should employ clearly defined metabolic exposures, standardized oral outcomes, repeated assessments, and longitudinal designs that analyze temporal relationships while considering confounding factors and modifications in concurrent care. Comparative interventions ought to assess periodontal treatment, weight reduction, nutritional strategies, and microbiome-targeted approaches through metrics such as disease progression, healing complications, tooth and implant longevity, and patient-reported outcomes. Investigations into adipokine signaling, epigenetic regulation, regenerative cellular techniques, platelet concentrates, and other innovative interventions must distinguish mechanistic effects from clinical benefits and identify the pertinent metabolic phenotype. Biomarker research should determine whether supplementary measurements enhance predictive accuracy or decision-making beyond current clinical and medical data. This research agenda maintains innovative proposals while explicitly outlining the evidence needed to evaluate each approach.

## Data Availability

No new data were created or analyzed in this study. Data sharing is not applicable to this article.

## Supplementary Materials

The following supporting information can be downloaded at: https://www.mdpi.com/article/10.3390/biomedicines14102255/s1, Supplementary Material S1: Complete Database Search Strategies.

## Abbreviations

The following abbreviations are used in this manuscript:

25(OH)D	25-hydroxyvitamin D
4NQO	4-Nitroquinoline 1-oxide
AAOMS	American Association of Oral and Maxillofacial Surgeons
AGE	advanced glycation end-product
AISI	Aggregate Index of Systemic Inflammation
ASA	American Society of Anesthesiologists
BMI	body mass index
CCL2	C-C motif chemokine ligand 2
CD36	cluster of differentiation 36
CI	confidence interval
CPAP	continuous positive airway pressure
CRP	C-reactive protein
CYP2R1	cytochrome P450 family 2 subfamily R member 1
CYP24A1	cytochrome P450 family 24 subfamily A member 1
CYP27A1	cytochrome P450 family 27 subfamily A member 1
CYP27B1	cytochrome P450 family 27 subfamily B member 1
DNA	deoxyribonucleic acid
DPP4	dipeptidyl peptidase-4
EFP	European Federation of Periodontology
FGR	FGR proto-oncogene, Src family tyrosine kinase
FTO	fat mass and obesity-associated gene
FPG	fasting plasma glucose
FYB1	FYN-binding protein 1
GIP	glucose-dependent insulinotropic polypeptide
GLP-1	glucagon-like peptide-1
GRADE	Grading of Recommendations Assessment, Development and Evaluation
H_2_S	hydrogen sulfide
HbA_1_c	glycated hemoglobin
HOMA-IR	homeostatic model assessment of insulin resistance
HOMA2-IR	homeostatic model assessment 2 of insulin resistance
hs-CRP	high-sensitivity C-reactive protein
IgA	immunoglobulin A
IL-1β	interleukin-1 beta
IL-6	interleukin-6
IL-8	interleukin-8
IL-10	interleukin-10
IL-17	interleukin-17
IR	insulin resistance
IRF4	interferon regulatory factor 4
ITI	International Team for Implantology
JAK2	Janus kinase 2
JMJD3	Jumonji domain-containing protein 3
LCK	lymphocyte-specific protein tyrosine kinase
LDL	low-density lipoprotein
LY86	lymphocyte antigen 86
m^6^A	N6-methyladenosine
MetS	metabolic syndrome
MMP	matrix metalloproteinase
MMP-1	matrix metalloproteinase-1
MRONJ	medication-related osteonecrosis of the jaw
NF-κB	nuclear factor kappa B
NHANES	National Health and Nutrition Examination Survey
OR	odds ratio
OSA	obstructive sleep apnea
oxLDL	oxidized low-density lipoprotein
P2RY13	purinergic receptor P2Y13
PPAR-γ	peroxisome proliferator-activated receptor gamma
RANKL	receptor activator of nuclear factor kappa-B ligand
RNA	ribonucleic acid
RUNX2	runt-related transcription factor 2
SII	Systemic Immune-Inflammation Index
SIRI	Systemic Inflammatory Response Index
STAT3	signal transducer and activator of transcription 3
STOP-BANG	Snoring, Tiredness, Observed apnea, high blood Pressure, Body mass index, Age, Neck circumference, and Gender
TNF-α	tumor necrosis factor alpha
TRAP	tartrate-resistant acid phosphatase
VEGF	vascular endothelial growth factor
VSC	volatile sulfur compound
WHO	World Health Organization
