# Supplementary material for: Obesity and Oral Health: Clinical Associations, Mechanistic Evidence, and Implications for Dental Care—A Narrative Review
Source: Biomedicines. 2026 Oct 6;14(10):2255. doi: 10.3390/biomedicines14102255 (PMC13701933; doi:10.3390/biomedicines14102255)
Supplement: Supplementary file 1 [file biomedicines-14-02255-s001.zip › biomedicines-4509473-supplementary.pdf]

Supplementary Material S1. Complete Database Search Strategies

PubMed/MEDLINE

Exposure block:

(
"Obesity"[Mesh] OR obes*[Title/Abstract]
OR overweight[Title/Abstract]
OR "Adiposity"[Mesh] OR adiposity[Title/Abstract]
OR "central obesity"[Title/Abstract]
OR "abdominal obesity"[Title/Abstract]
OR "visceral adiposity"[Title/Abstract]
OR "Metabolic Syndrome"[Mesh]
OR "metabolic syndrome"[Title/Abstract]
OR "Insulin Resistance"[Mesh]
OR "insulin resistance"[Title/Abstract]
OR hyperinsulin*[Title/Abstract]
)

Search 1: Periodontal disease

(
"Obesity"[Mesh] OR obes*[Title/Abstract]
OR overweight[Title/Abstract]
OR "Adiposity"[Mesh] OR adiposity[Title/Abstract]
OR "central obesity"[Title/Abstract]
OR "abdominal obesity"[Title/Abstract]
OR "visceral adiposity"[Title/Abstract]
OR "Metabolic Syndrome"[Mesh]
OR "metabolic syndrome"[Title/Abstract]
OR "Insulin Resistance"[Mesh]
OR "insulin resistance"[Title/Abstract]
OR hyperinsulin*[Title/Abstract]
)
AND
("Periodontal Diseases"[Mesh] OR periodont*[Title/Abstract] OR gingivitis[Title/Abstract] OR "alveolar bone loss"[Title/Abstract])

Search 2: Peri-implant disease and osseointegration

(
"Obesity"[Mesh] OR obes*[Title/Abstract]
OR overweight[Title/Abstract]
OR "Adiposity"[Mesh] OR adiposity[Title/Abstract]
OR "central obesity"[Title/Abstract]
OR "abdominal obesity"[Title/Abstract]
OR "visceral adiposity"[Title/Abstract]
OR "Metabolic Syndrome"[Mesh]
OR "metabolic syndrome"[Title/Abstract]
OR "Insulin Resistance"[Mesh]
OR "insulin resistance"[Title/Abstract]
OR hyperinsulin*[Title/Abstract]
)
AND

("Dental Implants"[Mesh] OR "Peri-Implantitis"[Mesh] OR peri-implant*[Title/Abstract] OR osseointegrat*[Title/Abstract] OR "implant failure"[Title/Abstract] OR "marginal bone loss"[Title/Abstract])

Search 3: Dental caries

(
"Obesity"[Mesh] OR obes*[Title/Abstract]
OR overweight[Title/Abstract]
OR "Adiposity"[Mesh] OR adiposity[Title/Abstract]
OR "central obesity"[Title/Abstract]
OR "abdominal obesity"[Title/Abstract]
OR "visceral adiposity"[Title/Abstract]
OR "Metabolic Syndrome"[Mesh]
OR "metabolic syndrome"[Title/Abstract]
OR "Insulin Resistance"[Mesh]
OR "insulin resistance"[Title/Abstract]
OR hyperinsulin*[Title/Abstract]
)
AND
("Dental Caries"[Mesh] OR caries[Title/Abstract] OR cariogenic[Title/Abstract])

Search 4: Salivary dysfunction, xerostomia, and halitosis

(
"Obesity"[Mesh] OR obes*[Title/Abstract]
OR overweight[Title/Abstract]
OR "Adiposity"[Mesh] OR adiposity[Title/Abstract]
OR "central obesity"[Title/Abstract]
OR "abdominal obesity"[Title/Abstract]
OR "visceral adiposity"[Title/Abstract]
OR "Metabolic Syndrome"[Mesh]
OR "metabolic syndrome"[Title/Abstract]
OR "Insulin Resistance"[Mesh]
OR "insulin resistance"[Title/Abstract]
OR hyperinsulin*[Title/Abstract]
)
AND
("Xerostomia"[Mesh] OR xerostomi*[Title/Abstract] OR "dry mouth"[Title/Abstract] OR saliv*[Title/Abstract] OR halitosis[Title/Abstract] OR "oral malodor"[Title/Abstract])

Search 5: Oral malignancy

(
"Obesity"[Mesh] OR obes*[Title/Abstract]
OR overweight[Title/Abstract]
OR "Adiposity"[Mesh] OR adiposity[Title/Abstract]
OR "central obesity"[Title/Abstract]
OR "abdominal obesity"[Title/Abstract]
OR "visceral adiposity"[Title/Abstract]
OR "Metabolic Syndrome"[Mesh]
OR "metabolic syndrome"[Title/Abstract]
OR "Insulin Resistance"[Mesh]
OR "insulin resistance"[Title/Abstract]

OR hyperinsulin*[Title/Abstract]
)
AND
("Mouth Neoplasms"[Mesh] OR "oral cancer"[Title/Abstract] OR "oral squamous cell carcinoma"[Title/Abstract] OR OSCC[Title/Abstract] OR "oral neoplasm*"[Title/Abstract] OR "oral tumor*"[Title/Abstract] OR "oral tumour*"[Title/Abstract])

Search 6: Jawbone biology and marrow alterations
(
"Obesity"[Mesh] OR obes*[Title/Abstract]
OR overweight[Title/Abstract]
OR "Adiposity"[Mesh] OR adiposity[Title/Abstract]
OR "central obesity"[Title/Abstract]
OR "abdominal obesity"[Title/Abstract]
OR "visceral adiposity"[Title/Abstract]
OR "Metabolic Syndrome"[Mesh]
OR "metabolic syndrome"[Title/Abstract]
OR "Insulin Resistance"[Mesh]
OR "insulin resistance"[Title/Abstract]
OR hyperinsulin*[Title/Abstract]
)
AND
(("Jaw"[Mesh] OR jaw[Title/Abstract] OR jawbone[Title/Abstract] OR mandib*[Title/Abstract] OR maxill*[Title/Abstract] OR alveolar[Title/Abstract]) AND ("Bone Density"[Mesh] OR "bone density"[Title/Abstract] OR "bone quality"[Title/Abstract] OR "bone marrow"[Title/Abstract] OR "marrow adiposity"[Title/Abstract] OR osteonecrosis[Title/Abstract] OR remodeling[Title/Abstract] OR remodelling[Title/Abstract]))

Search 7: Oral wound healing and surgical complications
(
"Obesity"[Mesh] OR obes*[Title/Abstract]
OR overweight[Title/Abstract]
OR "Adiposity"[Mesh] OR adiposity[Title/Abstract]
OR "central obesity"[Title/Abstract]
OR "abdominal obesity"[Title/Abstract]
OR "visceral adiposity"[Title/Abstract]
OR "Metabolic Syndrome"[Mesh]
OR "metabolic syndrome"[Title/Abstract]
OR "Insulin Resistance"[Mesh]
OR "insulin resistance"[Title/Abstract]
OR hyperinsulin*[Title/Abstract]
)
AND
("Wound Healing"[Mesh] OR "wound healing"[Title/Abstract] OR "tissue repair"[Title/Abstract] OR "socket healing"[Title/Abstract] OR "bone healing"[Title/Abstract] OR "postoperative complication*"[Title/Abstract])

Search 8: Oral and gut microbiome pathways
(
"Obesity"[Mesh] OR obes*[Title/Abstract]
OR overweight[Title/Abstract]

OR "Adiposity"[Mesh] OR adiposity[Title/Abstract]
OR "central obesity"[Title/Abstract]
OR "abdominal obesity"[Title/Abstract]
OR "visceral adiposity"[Title/Abstract]
OR "Metabolic Syndrome"[Mesh]
OR "metabolic syndrome"[Title/Abstract]
OR "Insulin Resistance"[Mesh]
OR "insulin resistance"[Title/Abstract]
OR hyperinsulin*[Title/Abstract]
)
AND
("Microbiota"[Mesh] OR microbiom*[Title/Abstract] OR microbiota[Title/Abstract] OR dysbiosis[Title/Abstract] OR "oral-gut"[Title/Abstract] OR "gut-oral"[Title/Abstract] OR "gut immune bone"[Title/Abstract])

Search 9: Mechanistic pathways
(
"Obesity"[Mesh] OR obes*[Title/Abstract]
OR overweight[Title/Abstract]
OR "Adiposity"[Mesh] OR adiposity[Title/Abstract]
OR "central obesity"[Title/Abstract]
OR "abdominal obesity"[Title/Abstract]
OR "visceral adiposity"[Title/Abstract]
OR "Metabolic Syndrome"[Mesh]
OR "metabolic syndrome"[Title/Abstract]
OR "Insulin Resistance"[Mesh]
OR "insulin resistance"[Title/Abstract]
OR hyperinsulin*[Title/Abstract]
)
AND
((inflamm*[Title/Abstract] OR "oxidative stress"[Title/Abstract] OR adipokin*[Title/Abstract] OR leptin[Title/Abstract] OR adiponectin[Title/Abstract] OR resistin[Title/Abstract] OR visfatin[Title/Abstract] OR epigenetic*[Title/Abstract] OR microRNA*[Title/Abstract] OR miRNA*[Title/Abstract] OR "vitamin D"[Title/Abstract]) AND (oral[Title/Abstract] OR periodont*[Title/Abstract] OR dental[Title/Abstract] OR jaw[Title/Abstract]))

Search 10: Lifestyle and shared determinants
(
"Obesity"[Mesh] OR obes*[Title/Abstract]
OR overweight[Title/Abstract]
OR "Adiposity"[Mesh] OR adiposity[Title/Abstract]
OR "central obesity"[Title/Abstract]
OR "abdominal obesity"[Title/Abstract]
OR "visceral adiposity"[Title/Abstract]
OR "Metabolic Syndrome"[Mesh]
OR "metabolic syndrome"[Title/Abstract]
OR "Insulin Resistance"[Mesh]
OR "insulin resistance"[Title/Abstract]
OR hyperinsulin*[Title/Abstract]
)
AND

((("Diet"[Mesh] OR diet*[Title/Abstract] OR sugar*[Title/Abstract] OR nutrition*[Title/Abstract] OR "Physical Activity"[Mesh] OR "physical activity"[Title/Abstract] OR stress[Title/Abstract] OR socioeconomic*[Title/Abstract] OR "oral hygiene"[Title/Abstract])) AND (oral[Title/Abstract] OR dental[Title/Abstract] OR periodont*[Title/Abstract]))

Search 11: Bariatric surgery and obstructive sleep apnea

(
  "Obesity"[Mesh] OR obes*[Title/Abstract]
  OR overweight[Title/Abstract]
  OR "Adiposity"[Mesh] OR adiposity[Title/Abstract]
  OR "central obesity"[Title/Abstract]
  OR "abdominal obesity"[Title/Abstract]
  OR "visceral adiposity"[Title/Abstract]
  OR "Metabolic Syndrome"[Mesh]
  OR "metabolic syndrome"[Title/Abstract]
  OR "Insulin Resistance"[Mesh]
  OR "insulin resistance"[Title/Abstract]
  OR hyperinsulin*[Title/Abstract]
)
AND
((("Bariatric Surgery"[Mesh] OR bariatric[Title/Abstract] OR "gastric bypass"[Title/Abstract] OR "sleeve gastrectomy"[Title/Abstract] OR "Sleep Apnea, Obstructive"[Mesh] OR "obstructive sleep apnea"[Title/Abstract] OR OSA[Title/Abstract])) AND (oral[Title/Abstract] OR dental[Title/Abstract] OR periodont*[Title/Abstract] OR xerostomi*[Title/Abstract] OR implant*[Title/Abstract]))

Scopus

Exposure block:
TITLE-ABS-KEY(
  obes* OR overweight OR adiposity
  OR "central obesity"
  OR "abdominal obesity"
  OR "visceral adiposity"
  OR "metabolic syndrome"
  OR "insulin resistance"
  OR hyperinsulin*
)

Search 1: Periodontal disease

TITLE-ABS-KEY(
  obes* OR overweight OR adiposity
  OR "central obesity"
  OR "abdominal obesity"
  OR "visceral adiposity"
  OR "metabolic syndrome"
  OR "insulin resistance"
  OR hyperinsulin*
)
AND
TITLE-ABS-KEY(periodont* OR gingivitis OR "alveolar bone loss")
AND PUBYEAR > 1999
AND PUBYEAR < 2027

Search 2: Peri-implant disease and osseointegration
TITLE-ABS-KEY(
obes* OR overweight OR adiposity
OR "central obesity"
OR "abdominal obesity"
OR "visceral adiposity"
OR "metabolic syndrome"
OR "insulin resistance"
OR hyperinsulin*
)
AND
TITLE-ABS-KEY("dental implant*" OR peri-implant* OR osseointegrat* OR "implant failure" OR "marginal bone loss")
AND PUBYEAR > 1999
AND PUBYEAR < 2027

Search 3: Dental caries
TITLE-ABS-KEY(
obes* OR overweight OR adiposity
OR "central obesity"
OR "abdominal obesity"
OR "visceral adiposity"
OR "metabolic syndrome"
OR "insulin resistance"
OR hyperinsulin*
)
AND
TITLE-ABS-KEY("dental caries" OR caries OR cariogenic)
AND PUBYEAR > 1999
AND PUBYEAR < 2027

Search 4: Salivary dysfunction, xerostomia, and halitosis
TITLE-ABS-KEY(
obes* OR overweight OR adiposity
OR "central obesity"
OR "abdominal obesity"
OR "visceral adiposity"
OR "metabolic syndrome"
OR "insulin resistance"
OR hyperinsulin*
)
AND
TITLE-ABS-KEY(xerostomi* OR "dry mouth" OR saliv* OR halitosis OR "oral malodor")
AND PUBYEAR > 1999
AND PUBYEAR < 2027

Search 5: Oral malignancy
TITLE-ABS-KEY(
obes* OR overweight OR adiposity
OR "central obesity"

OR "abdominal obesity"
OR "visceral adiposity"
OR "metabolic syndrome"
OR "insulin resistance"
OR hyperinsulin*
)
AND
TITLE-ABS-KEY("oral cancer" OR "oral squamous cell carcinoma" OR OSCC OR "oral neoplasm*" OR "oral tumor*" OR "oral tumour*")
AND PUBYEAR > 1999
AND PUBYEAR < 2027

Search 6: Jawbone biology and marrow alterations
TITLE-ABS-KEY(
obes* OR overweight OR adiposity
OR "central obesity"
OR "abdominal obesity"
OR "visceral adiposity"
OR "metabolic syndrome"
OR "insulin resistance"
OR hyperinsulin*
)
AND
TITLE-ABS-KEY((jaw OR jawbone OR mandib* OR maxill* OR alveolar) AND ("bone density" OR "bone quality" OR "bone marrow" OR "marrow adiposity" OR osteonecrosis OR remodeling OR remodelling))
AND PUBYEAR > 1999
AND PUBYEAR < 2027

Search 7: Oral wound healing and surgical complications
TITLE-ABS-KEY(
obes* OR overweight OR adiposity
OR "central obesity"
OR "abdominal obesity"
OR "visceral adiposity"
OR "metabolic syndrome"
OR "insulin resistance"
OR hyperinsulin*
)
AND
TITLE-ABS-KEY("wound healing" OR "tissue repair" OR "socket healing" OR "bone healing" OR "postoperative complication*")
AND PUBYEAR > 1999
AND PUBYEAR < 2027

Search 8: Oral and gut microbiome pathways
TITLE-ABS-KEY(
obes* OR overweight OR adiposity
OR "central obesity"
OR "abdominal obesity"
OR "visceral adiposity"

OR "metabolic syndrome"
OR "insulin resistance"
OR hyperinsulin*
)
AND
TITLE-ABS-KEY(microbiom* OR microbiota OR dysbiosis OR "oral-gut" OR "gut-oral" OR "gut immune bone")
AND PUBYEAR > 1999
AND PUBYEAR < 2027

Search 9: Mechanistic pathways
TITLE-ABS-KEY(
obes* OR overweight OR adiposity
OR "central obesity"
OR "abdominal obesity"
OR "visceral adiposity"
OR "metabolic syndrome"
OR "insulin resistance"
OR hyperinsulin*
)
AND
TITLE-ABS-KEY((inflamm* OR "oxidative stress" OR adipokin* OR leptin OR adiponectin OR resistin OR visfatin OR epigenetic* OR microRNA* OR miRNA* OR "vitamin D") AND (oral OR dental OR periodont* OR jaw))
AND PUBYEAR > 1999
AND PUBYEAR < 2027

Search 10: Lifestyle and shared determinants
TITLE-ABS-KEY(
obes* OR overweight OR adiposity
OR "central obesity"
OR "abdominal obesity"
OR "visceral adiposity"
OR "metabolic syndrome"
OR "insulin resistance"
OR hyperinsulin*
)
AND
TITLE-ABS-KEY((diet* OR sugar* OR nutrition* OR "physical activity" OR stress OR socioeconomic* OR "oral hygiene") AND (oral OR dental OR periodont*))
AND PUBYEAR > 1999
AND PUBYEAR < 2027

Search 11: Bariatric surgery and obstructive sleep apnea
TITLE-ABS-KEY(
obes* OR overweight OR adiposity
OR "central obesity"
OR "abdominal obesity"
OR "visceral adiposity"
OR "metabolic syndrome"
OR "insulin resistance"

OR hyperinsulin*
)
AND
TITLE-ABS-KEY((bariatric OR "gastric bypass" OR "sleeve gastrectomy" OR "obstructive sleep apnea" OR OSA) AND (oral OR dental OR periodont* OR xerostomi* OR implant*))
AND PUBYEAR > 1999
AND PUBYEAR < 2027

Web of Science Core Collection
Exposure block:
TS=(
obes* OR overweight OR adiposity
OR "central obesity"
OR "abdominal obesity"
OR "visceral adiposity"
OR "metabolic syndrome"
OR "insulin resistance"
OR hyperinsulin*
)

Search 1: Periodontal disease
TS=(
obes* OR overweight OR adiposity
OR "central obesity"
OR "abdominal obesity"
OR "visceral adiposity"
OR "metabolic syndrome"
OR "insulin resistance"
OR hyperinsulin*
)
AND
TS=(periodont* OR gingivitis OR "alveolar bone loss"): 2000-2026

Search 2: Peri-implant disease and osseointegration
TS=(
obes* OR overweight OR adiposity
OR "central obesity"
OR "abdominal obesity"
OR "visceral adiposity"
OR "metabolic syndrome"
OR "insulin resistance"
OR hyperinsulin*
)
AND
TS=("dental implant*" OR peri-implant* OR osseointegrat* OR "implant failure" OR "marginal bone loss"): 2000-2026

Search 3: Dental caries
TS=(
obes* OR overweight OR adiposity
OR "central obesity"

OR "abdominal obesity"
OR "visceral adiposity"
OR "metabolic syndrome"
OR "insulin resistance"
OR hyperinsulin*
)
AND
TS=("dental caries" OR caries OR cariogenic): 2000-2026

Search 4: Salivary dysfunction, xerostomia, and halitosis
TS=(
obes* OR overweight OR adiposity
OR "central obesity"
OR "abdominal obesity"
OR "visceral adiposity"
OR "metabolic syndrome"
OR "insulin resistance"
OR hyperinsulin*
)
AND
TS=(xerostomi* OR "dry mouth" OR saliv* OR halitosis OR "oral malodor"): 2000-2026

Search 5: Oral malignancy
TS=(
obes* OR overweight OR adiposity
OR "central obesity"
OR "abdominal obesity"
OR "visceral adiposity"
OR "metabolic syndrome"
OR "insulin resistance"
OR hyperinsulin*
)
AND
TS=("oral cancer" OR "oral squamous cell carcinoma" OR OSCC OR "oral neoplasm*" OR "oral tumor*" OR "oral tumour*"): 2000-2026

Search 6: Jawbone biology and marrow alterations
TS=(
obes* OR overweight OR adiposity
OR "central obesity"
OR "abdominal obesity"
OR "visceral adiposity"
OR "metabolic syndrome"
OR "insulin resistance"
OR hyperinsulin*
)
AND
TS=((jaw OR jawbone OR mandib* OR maxill* OR alveolar) AND ("bone density" OR "bone quality" OR "bone marrow" OR "marrow adiposity" OR osteonecrosis OR remodeling OR remodelling)): 2000-2026

Search 7: Oral wound healing and surgical complications

TS=(
  obes* OR overweight OR adiposity
  OR "central obesity"
  OR "abdominal obesity"
  OR "visceral adiposity"
  OR "metabolic syndrome"
  OR "insulin resistance"
  OR hyperinsulin*
)
AND
TS=("wound healing" OR "tissue repair" OR "socket healing" OR "bone healing" OR "postoperative complication*"): 2000-2026

Search 8: Oral and gut microbiome pathways

TS=(
  obes* OR overweight OR adiposity
  OR "central obesity"
  OR "abdominal obesity"
  OR "visceral adiposity"
  OR "metabolic syndrome"
  OR "insulin resistance"
  OR hyperinsulin*
)
AND
TS=(microbiom* OR microbiota OR dysbiosis OR "oral-gut" OR "gut-oral" OR "gut immune bone"): 2000-2026

Search 9: Mechanistic pathways

TS=(
  obes* OR overweight OR adiposity
  OR "central obesity"
  OR "abdominal obesity"
  OR "visceral adiposity"
  OR "metabolic syndrome"
  OR "insulin resistance"
  OR hyperinsulin*
)
AND
TS=((inflamm* OR "oxidative stress" OR adipokin* OR leptin OR adiponectin OR resistin OR visfatin OR epigenetic* OR microRNA* OR miRNA* OR "vitamin D") AND (oral OR dental OR periodont* OR jaw)): 2000-2026

Search 10: Lifestyle and shared determinants

TS=(
  obes* OR overweight OR adiposity
  OR "central obesity"
  OR "abdominal obesity"
  OR "visceral adiposity"
  OR "metabolic syndrome"
  OR "insulin resistance"

OR hyperinsulin*
)
AND
TS=((diet* OR sugar* OR nutrition* OR "physical activity" OR stress OR socioeconomic* OR "oral hygiene") AND (oral OR dental OR periodont*)): 2000-2026

Search 11: Bariatric surgery and obstructive sleep apnea
TS=(
obes* OR overweight OR adiposity
OR "central obesity"
OR "abdominal obesity"
OR "visceral adiposity"
OR "metabolic syndrome"
OR "insulin resistance"
OR hyperinsulin*
)
AND
TS=((bariatric OR "gastric bypass" OR "sleeve gastrectomy" OR "obstructive sleep apnea" OR OSA) AND (oral OR dental OR periodont* OR xerostomi* OR implant*)): 2000-2026
